# Small RNAs, DNA methylation and transposable elements in wheat

**DOI:** 10.1186/1471-2164-11-408

**Published:** 2010-06-29

**Authors:** Dario Cantu, Leonardo S Vanzetti, Adam Sumner, Martin Dubcovsky, Marta Matvienko, Assaf Distelfeld, Richard W Michelmore, Jorge Dubcovsky

**Affiliations:** 1 Department of Plant Sciences, University of California Davis, One Shields Ave, Davis, CA, USA; 2 Grupo de Biotecnología y Rec. Genéticos, INTA EEA Marcos Juárez, Ruta 12 S/N, (2580) Marcos Juárez, Córdoba, Argentina; 3 Department of Physics, University of California Davis, One Shields Ave, Davis, CA, USA; 4 Genome Center, University of California Davis, One Shields Ave, Davis, CA, USA

## Abstract

**Background:**

More than 80% of the wheat genome is composed of transposable elements (TEs). Since active TEs can move to different locations and potentially impose a significant mutational load, their expression is suppressed in the genome via small non-coding RNAs (sRNAs). sRNAs guide silencing of TEs at the transcriptional (mainly 24-nt sRNAs) and post-transcriptional (mainly 21-nt sRNAs) levels. In this study, we report the distribution of these two types of sRNAs among the different classes of wheat TEs, the regions targeted within the TEs, and their impact on the methylation patterns of the targeted regions.

**Results:**

We constructed an sRNA library from hexaploid wheat and developed a database that included our library and three other publicly available sRNA libraries from wheat. For five completely-sequenced wheat BAC contigs, most perfectly matching sRNAs represented TE sequences, suggesting that a large fraction of the wheat sRNAs originated from TEs. An analysis of all wheat TEs present in the *Triticeae *Repeat Sequence database showed that sRNA abundance was correlated with the estimated number of TEs within each class. Most of the sRNAs perfectly matching miniature inverted repeat transposable elements (*MITEs*) belonged to the 21-nt class and were mainly targeted to the terminal inverted repeats (TIRs). In contrast, most of the sRNAs matching class I and class II TEs belonged to the 24-nt class and were mainly targeted to the long terminal repeats (LTRs) in the class I TEs and to the terminal repeats in *CACTA *transposons. An analysis of the mutation frequency in potentially methylated sites revealed a three-fold increase in TE mutation frequency relative to intron and untranslated genic regions. This increase is consistent with wheat TEs being preferentially methylated, likely by sRNA targeting.

**Conclusions:**

Our study examines the wheat epigenome in relation to known TEs. sRNA-directed transcriptional and post-transcriptional silencing plays important roles in the short-term suppression of TEs in the wheat genome, whereas DNA methylation and increased mutation rates may provide a long-term mechanism to inactivate TEs.

## Background

The genome of hexaploid wheat (2n = 6X = 42; genomes AABBDD) is one of the largest in the grass family. The 2C DNA content of hexaploid wheat is 33.1 pg, about 37 and 165 times the genome size of rice (*Oryza sativa*) and *Arabidopsis thaliana*, respectively [1]. Based on DNA re-association studies the non-repetitive DNA fraction is estimated to be about 17% of the wheat genome [2], or hypothesized to be as low as 1% based on available sequence data analysis and genome size in relation to other plant genomes [3]. The repetitive, non-genic regions of wheat, as in many plant genomes, primarily consist of transposable elements (TEs) [4-7] and to a much lesser extent of pseudogenes [8-11]. During the past few years, about 1,500 *Triticeae *TE sequences have been discovered and deposited in the database for *Triticeae *repeats (TREP; http://wheat.pw.usda.gov/ITMI/Repeats).

First discovered by Barbara McClintock (1950) in maize, TEs have been reported to be present in all genomes analyzed, with similarities even among life kingdoms [12]. TEs are discrete sequences in the genome that can multiply and/or move within a host genome [13]. Class I TEs, which include long terminal repeat (LTR) retrotransposons and non-LTR transposons, are transcribed into mRNA that is subsequently reverse transcribed into DNA by a reverse transcriptase. Class II TEs, which are DNA transposons, including terminal inverted repeats (TIR) transposons, miniature inverted repeat transposable elements (*MITEs*) and *Helitrons*, move as DNA molecules that are excised from a genomic position and integrate elsewhere [14]. TEs are now recognized as important contributors to genomic organization and as major drivers of genome evolution. Centromeric and pericentromeric regions mainly consist of TEs [15-17], which may play an important role in centromeric stability and heterochromatin maintenance [18,19]. Induced activation of TEs resulted in altered chromosome segregation and meiotic disruption in mouse [20], loss of sister chromatid cohesion in yeast [21] and loss of centromere condensation in *A. thaliana *[22].

Active TEs constitute a major source of mutations in the genome. Transposition of a TE can result in altered gene expression [23-30], generation of novel regulatory networks [31], gene deletions [32,33], gene duplications [34], increases in genome size [6,35,36], illegitimate recombination [37] and chromosome breaks and rearrangements [38,39]. Because of the potential harmful effects of active TEs, the expression of most TEs in the genome is suppressed so that, even if whole and capable of autonomous transposition, most TEs remain silent throughout the plant's life cycle [19]. Only few naturally active TEs have been identified so far [12,40]. Nonetheless, TE-derived sequences are abundant in wheat cDNA libraries [41] and activation of TEs has been observed under conditions of biotic and abiotic stresses [42,43]. TE expression is silenced both at transcription and after transcription through epigenetic mechanisms [19].

TEs can be transcriptionally silenced by DNA methylation and repressive chromatin formation, involving modifications of histone tails and altered chromatin packing [12,44,45]. Post-transcriptional silencing of TEs is achieved by the degradation of TE transcripts by RNA-degrading complexes [12,46-48]. Small non-coding RNAs (sRNAs), generated when double-stranded RNA (dsRNA) is cleaved by proteins belonging to the Dicer family, guide the sequence-specific silencing after transcription [49]. sRNAs are also involved in DNA methylation of homologous DNA sequences in the nucleus (RNA-directed DNA methylation) and heterochromatin formation, guiding the silencing of TE at the transcriptional level [50,51]. The function of sRNAs is related to their length: if 21-nt long, silencing is post-transcriptional, whereas if 24-nt long, silencing is mediated by RNA-dependent DNA methylation and heterochromatin maintenance [19,51]. TEs are mobilized in *Caenorhabditis elegans *mutants that are defective in RNAi [52,53] and in mutants of *A. thaliana *that are deficient in DNA methylation and chromatin structure regulation [45,54-56]. Beside TE-silencing, the sRNAs are involved in a wide variety of biological phenomena, ranging from developmental processes to responses to biotic and abiotic stresses [57].

High-throughput sequencing has greatly facilitated the analysis of sRNA sequences. Massively-parallel sequencing platforms allow the identification of hundreds of thousands of sRNAs in any organism [58-66]. Profiles of sRNA collected from 22 species of higher plants, including wheat, are now publically available http://smallrna.udel.edu/.

In wheat, fast rates of TE insertion and deletion result in rapid turnover of intergenic regions, which can affect neighbouring genes [67]. This fast mutation frequency, together with the high tolerance to mutations of a polyploid genome, accounts for the genomic dynamism and adaptability of wheat [67]. Regulation of TE expression in the wheat genome has not been studied in detail. In this study, we report the analysis of the different classes of sRNAs originated from the different known classes of TEs in wheat, their target regions within the repetitive elements, and their impact on the methylation patterns of the targeted regions.

## Results

### Sequencing of sRNAs and comparison to extant public libraries

To investigate the relationship between sRNAs and TEs in wheat, we constructed an sRNA library from leaves of *T. aestivum *and developed a database that included our library plus the three libraries of sRNAs from *T. aestivum *that were publicly available at http://smallrna.udel.edu/. We sequenced 1,074,691 sRNAs (TAE4 library; GEO accession: GSM548032), which were then combined with 3,570,129 sRNAs from *T. aestivum *leaves (TAE1 library), as well as 2,916,955 and 2,968,383 sRNAs from *T. aestivum *healthy (TAE2 library) and *Fusarium*-infected spikelets (TAE3 library), respectively (Additional File 1 Table S1; the python program "dbmanager.py" written for the sRNA database setup is in Additional File 2). The resulting database is composed of 10,530,158 sRNA sequences (3,755,852 distinct sRNAs), with a bimodal size distribution with peaks at 21-nt (17.7 ± 4.6%) and 24-nt (28.7 ± 9.2%). Since libraries were from different tissues and developmental stages some variability was observed in the abundance of the 21 and 24-nt classes, which are summarized in the Additional File 1 Table S1.

The hexaploid wheat used to construct the TAE4 sRNA library expressed an RNAi construct under the 35S promoter that targeted the endogenous *NO APICAL MERISTEM *(*NAM*) gene [68]. The presence of this RNAi transgene caused a 40% reduction in expression of the target genes as measured by quantitative RT-PCR [68]. Out of 1,074,691 sRNAs in the TAE4 library, 4,105 (88.1% 21-nt and 6.3% 24-nt) perfectly matched the targeted NAM sequence in the RNAi construct reflecting the efficacy of the silencing construct (Additional File 1 Table S3). No sRNA from the other libraries made from non-transgenic materials matched the target *NAM *gene. We cannot rule out the possibility that the RNAi may cause other effect on the sRNA population.

### Distribution of sRNA counts within annotated BAC sequences

To explore the distribution of sRNAs in relation to both the sequences from which they originated and their potential targets, we mapped the sRNAs from the four libraries present in our database onto five completely-sequenced genomic regions, three from tetraploid wheat (*T. turgidum*) and two from hexaploid wheat (*T. aestivum*). Five entirely annotated genomic regions, EU835198 (314,057 bp) [69], DQ871219 (245,486 bp) [68], EF540321 (291,163 bp) [70], EF567062 (137,614 bp) [71], and DQ537335 (292,102 bp) [72] obtained by sequencing 10 bacterial artificial chromosomes (BACs) were chosen. Altogether, these regions (1,280,422 bp) include 190 (63% of the total genomic region analyzed) TEs (65% class I and 35% class II) and 26 (6% of the total genomic region analyzed) genes. The gene density of these genomic regions ranges from 1 gene per 34 kb in EF567062 to 1 gene per 63 kb in EU835198, similar to that observed in other wheat gene-rich regions [6,73-75].

**Figure 1:**
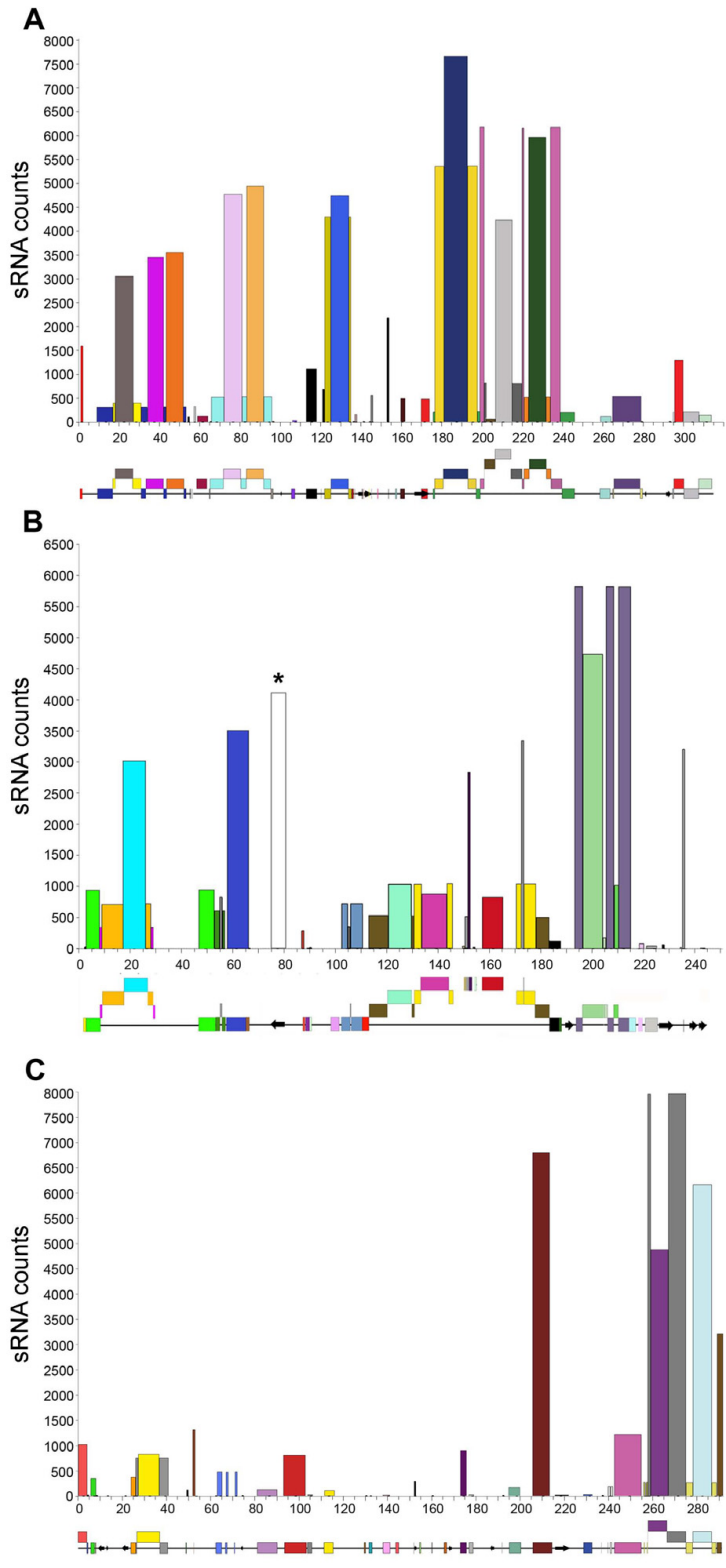
**sRNA counts over annotated genomic regions**. Bar graphs representing the total counts of sRNA that perfectly matched annotated TEs and genes in EU835198 (A), DQ871219 (B), and EF540321 (C). sRNA counts and size of the annotated locus to which the sRNAs match correspond to bar height and bar width, respectively. A graphical representation of the structure of the genomic regions is provided below each bar graph. TEs are shown as colored boxes and genes as arrows. More recent insertions of repetitive elements are shown as boxes nested above the elements into which they were inserted. Boxes of the same colour and at the same level are part of the same element. In Figure 1B the bar with asterisk corresponds to the total counts of sRNAs from library TAE4 matching the RNAi target (*TaNAM*) gene.

A scrolling window analysis (Additional Files 3 and 4) was done to identify all sRNAs that perfectly matched the genomic sequences. Table 1 shows the distributions of the counts of sRNA mapping to the annotated TEs and genes in the three *T. turgidum *genomic regions, EU835198, DQ871219, and EF540321 (Figures 1A to 1C respectively) and the two *T. aestivum *sequences EF567062 and DQ537335. TEs and gene coordinates within each of the five regions and the relative sRNA counts are reported in Additional File 1 Tables S2-6. The number of perfectly matching sRNAs ranged from 71,868 (0.23 sRNA counts/bp for DQ871219) to 17,447 sRNAs (0.13 sRNA counts/bp for EF567062) (Table 1). Similar profiles of sRNA distribution on TEs and gene-encoding regions was observed in the three genomic regions from *T. turgidum *and the two of *T. aestivum*: ninety three percent of the total sRNAs (92% in *T. turgidum*; 94% in *T. aestivum*) that matched these genomic regions matched TE sequences (average 0.29 sRNA counts/bp), whereas only 0.07% (0.05% in *T. *turgidum; 0.1% in *T. *aestivum) matched the gene-encoding regions (average 0.001 sRNA counts/bp, excluding the sRNA that matched the *NAM *RNAi region in the GPC_RNAi library). A statistical comparison of the sRNA/bp in TEs and gene regions showed highly significant differences (*P *< 0.001).

**Table 1:** Distribution of sRNA matches between transposable elements and genes in three annotated genomic regions

		Total			Transposable elements			Genes		
**Region**	**Organism**	**Length (bp)**	**sRNA counts**	**sRNA counts/bp**	**Length (bp)**	**sRNA counts**	**sRNA counts/bp**	**Length (bp)**	**sRNA counts**	**sRNA counts/bp**

EU835198	*T. turgidum*	314,057	71,868	0.23	220,511	70,808	0.32	18,298	8	0.00
DQ871219	*T. turgidum*	245,486	43,789	0.18	144,831	37,374	0.26	15,324	59 *	0.00
EF540321	*T. turgidum*	291,163	43,074	0.15	131,089	38,216	0.29	16,412	13	0.00
EF567062	*T. aestivum*	137,614	17,447	0.13	83,355	17,008	0.20	14,335	30	0.00
DQ537335	*T. aestivum*	292,102	41,761	0.14	231,351	39,091	0.17	11,096	32	0.00

* 54 of these match to a single gene annotated as a predicted leucine rich repeat gene.

Within the TEs, class I and class II TEs showed a similar sRNA density (class I: 0.24 sRNA counts/bp; class II: 0.26 sRNA counts/bp). However, 74% of the sRNAs that matched the class II TEs, correspond to the miniature inverted repeats transposable elements (MITEs), which account for only 4.2% of the class II TEs and, thus, have a significantly higher sRNA density than class I and the rest of the class II TEs (4.65 sRNA counts/bp; *P *= 7.0 × 10 ^-7^).

Many of the TEs present in the analyzed regions are organized in nested structures that include up to four layers of nested insertions, with the relative position in the nested structure providing an estimate of their relative insertion times [6,36]. We observed a significantly higher number of sRNAs matching the TEs that were at the top of the nested structures compared to the number of sRNAs that were associated with elements at the lower, more ancient layers of the nested structures (*P *= 0.047).

### Comparison between the predicted methylation pattern of TEs and genic regions

Because TEs accounted for most of the sRNAs in the genomic regions analyzed and transcriptional silencing, including cytosine methylation, is directed by sRNAs [76], we hypothesized that a higher level of cytosine methylation would be present in the repetitive elements than in the single-copy regions associated with genes, such as untranslated regions (UTRs) and introns. We used an *in silico *approach that took advantage of the different mutation rates of methylated C to infer the methylated regions [77]. Methylated cytosines display higher frequency of mutation than non-methylated cytosines because of a 10-fold increase in transition rate due to the passive deamination of methylated cytosine into thymine [78]. In plants, C methylation can occur not only in CG di-nucleotides , as observed in mammals, but also in CHG and, less frequently, in CHH tri-nucleotides, where H is A, T, or C [79].

We were able to use this approach only for the 80 kb *VRN2 *region, for which we had orthologous sequences from the *T. monococcum *A ^m ^genome BAC AY485644 and the *T. turgidum *A genome BAC EF540321 (these two genomes are >96% identical and diverged approximately 1 million years ago from each other [67]). The two orthologous regions were aligned and compared to count the number of mutations in potentially methylated sites (PMS).

**Figure 2:**
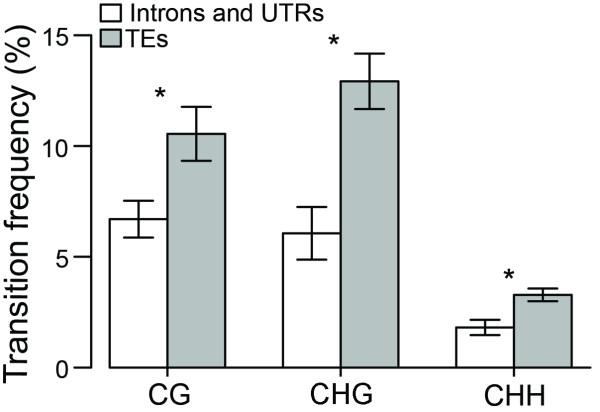
**Transition frequencies in potentially methylated sites (CG, CHG, and CHH)**. White bars represent introns and untranslated (UTR) gene regions. Exons were excluded. Gray bars represent transposable elements (TEs). Frequencies were calculated using the program "Cmet scan" (Additional File 5). Bars represent standard errors of the means.

**Figure 3:**
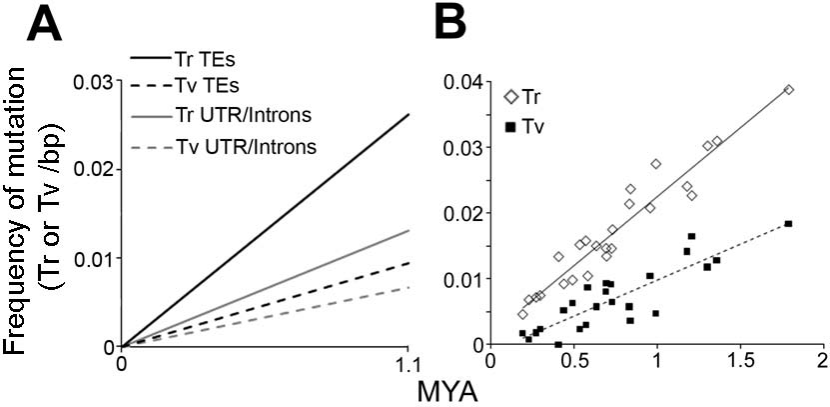
**Rate of transition and transversion in UTR/Introns and TEs**. (A) Number of transitions (Tr) and transversion (Tv) per bp that occurred in the orthologous *VRN2 *regions in *T. monococcum *and *T. turgidum *A genome during their 1.1 million year (MYA) of divergence. (B) Scatterplot representing the relation between the estimated insertion time (MYA) of LTR retrotransposons present in the *VRN2 *locus of *T. monococcum *and *T. turgidum *(LTRs are identical at the time of insertion) and the frequency of Tr and Tv in the LTRs. The slopes of the estimated linear trends represent the transition and transversions rates.

We developed a computer program, "Cmet scan" (Additional File 5), to classify all the C and G sites as CG, CHG, and CHH and to count the transitions that occurred at these sites between the two aligned sequences. The percent of mutations in PMS is used here as a proxy to infer the prevalence of methylation in a genomic region. Exonic sequences were excluded from the analysis to minimize the effect of selection on mutation frequencies. Our analysis confirmed that the overall frequency of mutations (transitions and transversions) was higher in PMS than in non-PMS sites. The observed number of mutations per nucleotide was approximately 20-fold and 11-fold higher in PMS than in non-PMS sites in TEs and in intronic and untranslated regions, respectively. The average percentage of CG, CHG, and CHH sites that underwent transitions was 2-fold higher (*P *< 0.05) in TEs than in intronic and untranslated regions (Figure 2). This difference between TEs and intronic and untranslated regions was also reflected in a higher transition to transversion ratio in the TEs (2.8) relative to the intronic and untranslated regions (1.9). This difference was the result of a higher transition frequency in the TEs versus the intronic and untranslated regions (*P *= 0.002; Figure 3A), with no apparent difference in the frequency of transversion (*P *= 0.59).

To analyze how the different rates of mutation affected the sequence divergence of the repetitive elements with time, we calculated the divergence rate per MYA using a previous estimate of 1.1 MYA of divergence between the *T. monococcum *and wheat A genome in the *VRN2 *region [67]. This estimate was based on the Kimura two-parameter method (K2P) [6,36,80] and a mutation rate of 5.5 x 10 ^-9 ^substitutions per synonymous site per year for the intronic and low copy number regions (10,397 aligned bp, 76 transitions and 51 transversions). Analysis of the orthologous TEs adjusted for the same divergence time resulted in a mutation rate of 1.67 x 10 ^-8 ^substitutions per synonymous site per year.

Using the previous rate, we calculated the hypothetical insertion times of the LTR retrotransposons in both of the homologous regions of *T. monococcum *and *T. turgidum*, assuming that the two LTRs are identical copies of the same template at the time of insertion [36]. Estimates of insertion time were possible for 24 elements with intact pairs of LTRs, 7 in *T. turgidum *and 17 in *T. monococcum *(Additional File 1 Table S8). Similarly to the comparison between orthologous transposable elements in the *VRN2 *the transition rate was approximately 2-fold higher than the transversion rate.

### Distribution of sRNAs among TEs

**Figure 4:**
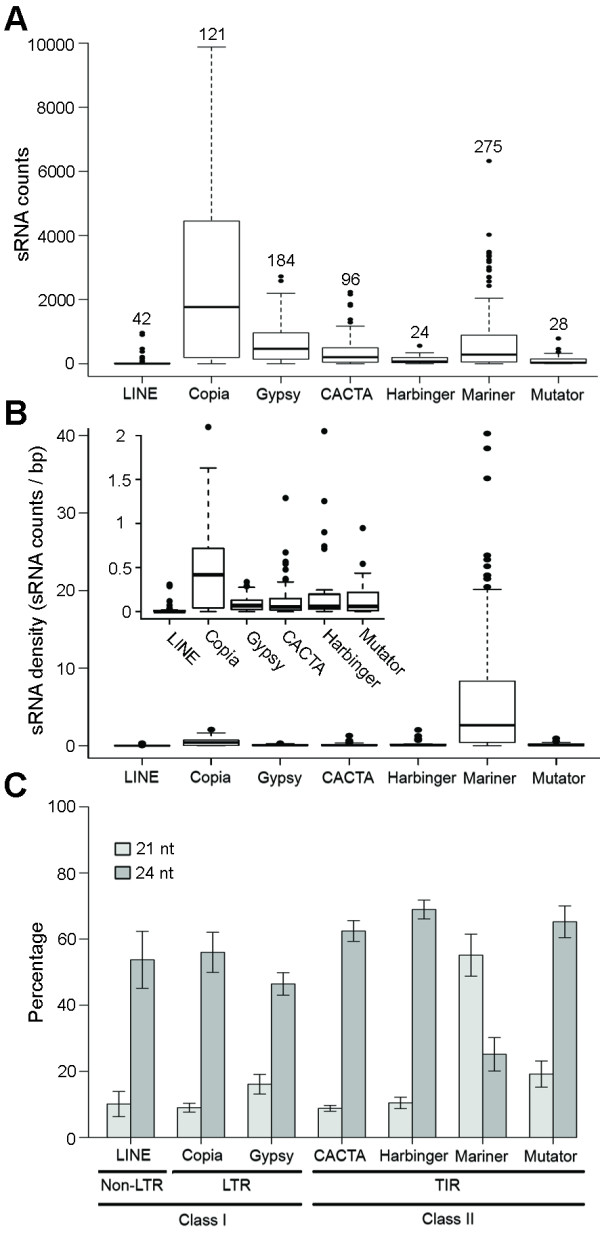
**Distribution of sRNAs among TEs**. (A) sRNA counts. Box plots represent the distribution of the total counts of sRNA perfectly matching wheat TEs of the seven major superfamilies deposited in the TREP database. Numbers above the whiskers represent the number of TREP elements within each superfamily considered. (B) sRNA density. Same data as before but adjusted by the size of the TEs. The inset represents all superfamiles excluding *Mariner *and an expanded scale. (C) Bar graph representing the percentage of 21 and 24 nucleotide sRNAs out of the total number that perfectly matched each superfamily of wheat TEs. Bars represent standard errors of the means.

To extend our study to more TEs than those present in the five sequenced genomic regions, we analyzed the targets of the sRNA among the repetitive DNA sequences deposited in the *Triticeae *Repeat Sequence Database (TREP, http://wheat.pw.usda.gov/ITMI/Repeats/). The complete TREP database (Release 10, July 2008) contains sequences for 1,562 *Triticeae *TEs, of which 1,005 are complete elements. From the complete elements, we selected 918 that belong to the genera *Triticum *and *Aegilops*, and, among those, the 877 that include no ambiguous base calls (e.g., N). The results from the query of the sequences of these 877 elements for perfect matches to our sRNA database are detailed in Additional File 1 Table S7 and summarized graphically in Figure 4 (major TE superfamilies) and Additional File 1 Figure S1 (major TE families within major superfamilies). *Copia *elements showed the highest sRNA counts, about 2-fold higher than *Mariner *TEs and about 3-fold higher than *Gypsy *TEs. Of the sRNA perfectly matching *Copia *TEs, 93% matched *Angela *(62%) and *WIS *(31%) TEs, which also showed the highest median sRNA counts per element (*Angela *3,228 and *WIS *3,648). Among the Gypsy TEs, the ones with highest median sRNA counts where *Wham *(972) and *Sabrina *(925), but together accounted only for 38% of the total sRNA that matched *Gypsy *TEs, reflecting the higher diversity of abundant *Gypsy *TEs. *Caspar*, *Clifford*, *Hamlet*, and *Jorge *TEs accounted for 69% of the sRNAs perfectly matching *CACTA *TEs. Among *Mariner *TEs, *Thalos MITEs *showed the highest median sRNA count per element (614) and alone accounted for 44% of the sRNA matching its superfamily.

To see if the differences in sRNA counts were correlated with the abundance of the elements, we estimated the number of TEs from the different classes in the wheat genome using the TREP database. In the complete TREP database, the class I LTR retrotransposons *Copia *(14%) and *Gypsy *(21%), and the class II elements *Mariner *(32%) and *CACTA *(11%) are the most abundant TE superfamilies, accounting together for about 80% of all TEs (Table 2). Accordingly, the TEs belonging to these superfamilies displayed the largest number of sRNAs counts, accounting for 97% of the 644,720 sRNAs that perfectly matched the 877 selected TE sequences. These perfect matches included 2,474 sRNAs per *Copia *element in TREP, 614 per *Gypsy*, 377 sRNAs per *CACTA*, and 655 sRNAs per *Mariner*, suggesting an excess of sRNA matching *Copia *elements. The correlation between element abundance in the TREP database and total sRNA counts per element was *r *= 0.63 (*P = *0.129), and increased markedly to *r *= 0.99 (*P *< 0.0001) when *Copia *TEs were excluded from the analysis.

**Table 2:** Total sRNA counts and element abundance in 21 annotated BACs, TREP database and blastn hits in T. aestivum NCBI EST collection

	Total sRNA (%)	21 BAC counts (%)	TREP counts (%)	EST hits (%)
**Class I**				
***Gypsy***	19.9	38.2	23.9	43.1
***Copia***	57.9	19.4	15.7	26.2
***LINE***	0.3	4.3	5.4	1.3
**Class II**				
***CACTA***	3.6	14.3	12.5	9.3
***Harbinger***	0.3	0.9	3.1	0.9
***Mutator***	0.3	0.9	3.6	0.7
***Mariner***	17.7	22.0	35.7	18.5

Data are expressed as percentage of the total values per column.

To determine whether *Copia *TEs were underrepresented in the TREP database (121 TEs) compared to other TE superfamilies, such as *Mariner *(274 TEs) and *Gypsy *(184), we estimated the abundance of TEs in *Triticum *by analyzing 21 annotated BACs (NCBI; http://www.ncbi.nlm.nih.gov/; Table 2). In the 3,356,076 bp analyzed, *Gypsy *TEs were the most abundant (205 instances) followed by *Mariner *(118) and *Copia *(104). This frequency distribution was very similar to the one found in the TREP database (*r *= 0.79, *P *= 0.033). The higher abundance of *Gypsy *relative to *Copia *was confirmed in a 1X shotgun sequencing of the complete hexaploid wheat genome (http://www.cerealsdb.uk.net/search_reads.htm; K.J. Edwards, personal communication). In summary, all the different estimates of TE copy number confirmed that at least six times more sRNAs matched individual *Copia *than *Gypsy *elements.

We also studied the representation of the different TE superfamilies in the NCBI collection of *T. aestivum *ESTs (Table 2). The number of TEs in the EST libraries is expected to be proportional to their abundance in the RNA population and, thus, related to their transcriptional activity. Database searches were carried out using the blastn search tool with an E-value threshold of 1e ^**-10 **^for *CACTA*, *Copia*, *LINE*, *Gypsy*, and of 1e ^**-6 **^for *Mariner*, *Harbinger*, and *Mutator *TEs to account for the smaller sizes of the latter. Correlations between sRNA matches and TE representation in the EST database showed a similar pattern to what was observed in the correlations with the genomic data: overall, sRNA counts significantly correlated with BLASTn hits in the EST library only if *Copia *were excluded from the analysis (with *Copia*: *r = *0.64, *P *= 0.12; without *Copia*: *r *= 0.91, *P <*0.01).

When the total number of counts was replaced by the counts per bp (match density), the *Stowaway *MITEs and the *Mariner *superfamily showed the highest density of sRNA counts (average 5.6 sRNA counts/bp), which was about 10-fold higher than *Copia *TEs, 65-fold higher than *Gypsy *TEs, and 47-fold higher than CACTA TEs (Figure 4B). In addition to the highest match density, the *Mariner *TEs presented a different pattern of sRNA sizes than the other groups (Figure 4C). *Mariner *TEs were the only class for which 21-nt sRNAs (54%) matches exceeded 24-nt sRNAs matches (27%), whereas for all other classes of TEs 24-nt matches were higher than 21-nt matches.

### Distribution of sRNAs within TEs

**Figure 5:**
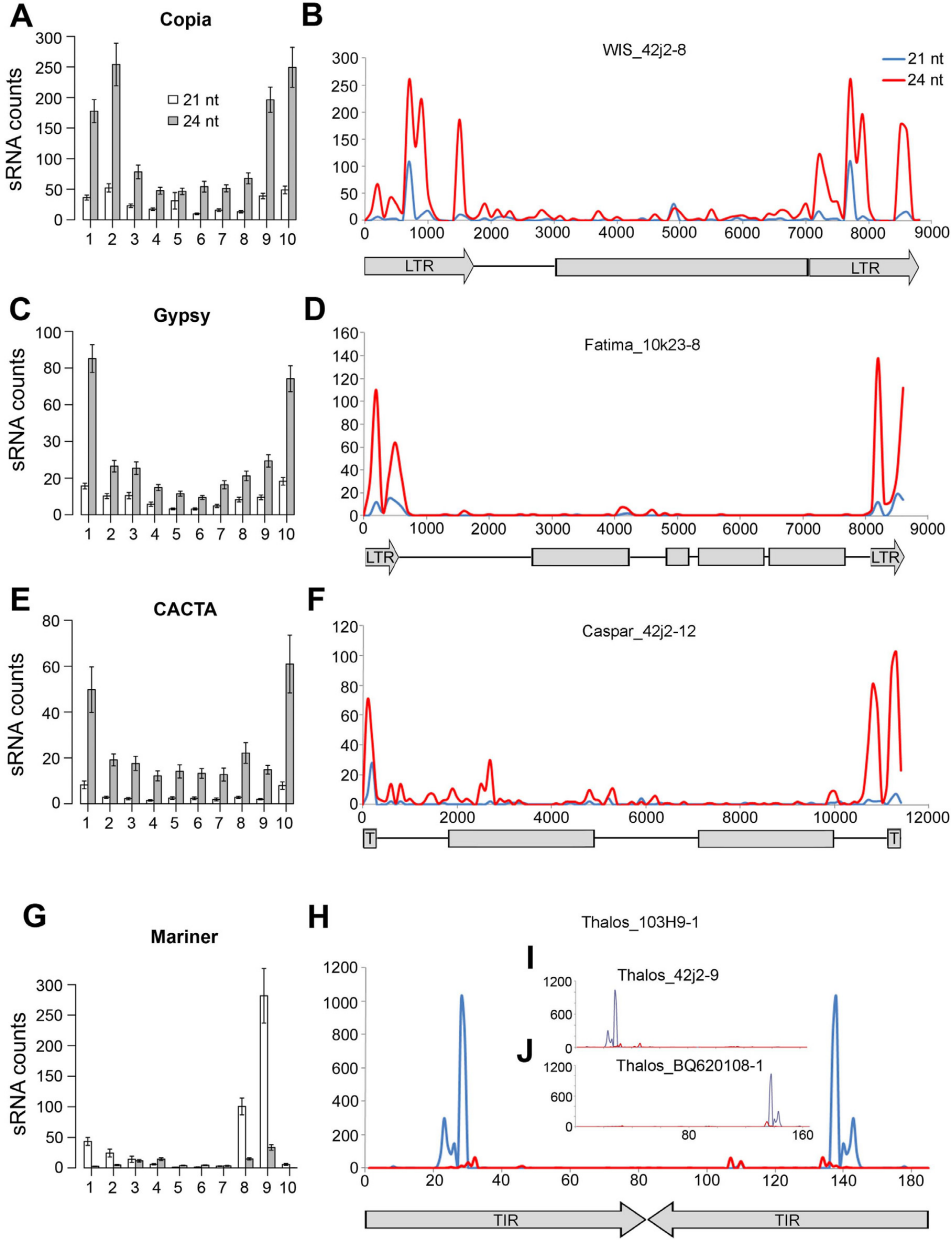
**Distribution of sRNAs within TEs**. The nucleotide sequence of each element of the *Copia*, *Gypsy*, *CACTA *and *Mariner *superfamilies present in the complete TREP database were divided in ten fractions, each representing 10% of the total element. (A, C, E, G). Bar graphs represent the mean number of total sRNAs that perfectly matched each of the ten fractions within each TE superfamily. Bars represent standard errors of the means. (B, D, F, H) Distribution of perfectly matching sRNAs in representative elements from each TE superfamily. A graphical representation of the elements is provided below each graph; arrows and boxes correspond to repeats and open reading frames, respectively. (I, J) Distribution of perfectly matching sRNAs in *MITEs *with mutations in the 5' (I) and 3' (J) regions.

To explore whether sRNAs preferentially matched specific regions of each TE, we divided the nucleotide sequence of each element of the *Copia*, *Gypsy*, *CACTA *and *Mariner *superfamilies present in the TREP database, into ten equal sections and determined the number of sRNAs that perfectly matched each section (Figure 5A,C, E, G). About 70% of the 24-nt sRNA matching the large LTR TEs was concentrated in the first and last 10% (*Gypsy*) and 20% (*Copia*) of these elements, corresponding to the LTRs. This observation was supported by the analysis of single elements (Figure 5B, D). Chi square tests averaging the duplicated LTR classes showed that the distribution of sRNA matches over the ten intervals differed significantly from a uniform distribution (df = 7 for *Copia *anddf = 8 for *Gypsy*; *P *< 0.0001).

Complete *CACTA *elements are flanked by short terminal repeats (TIRs) that terminate in the *CACTA *motifs [81]. *CACTA *elements also contain sub-terminal repeats in direct and inverted orientation (TRs). Sub-terminal repeats typically lack sequence conservation between different families. About 50% of the sRNAs matching the *CACTA *elements matched the first and last 10% of these elements, which was also significantly different from a uniform distribution (sections 1 and 10 were averaged, df = 8, *P *< 0.0001; Figure 5E). The distal 10% corresponds to the TRs and adjacent sequences as suggested by the analysis of individual *Caspar *TEs (Figure 5F).

Wheat *Stowaway MITEs *represent the largest group within the *Mariner *superfamily. They are small (50-500 bp), non-autonomous elements that end in well conserved TIRs that comprise the majority of their structure. About 93% of the sRNAs matching this group of TEs are targeted to the TIRs. The higher frequency of sRNA in the 3'-TIR is likely due to a larger number of 5'-truncated elements in the TREP database (Figure 5G-J). The analysis of the individual *MITE *Stowaway *Thalos *103H9-1 (composed of only two TIRs) shows a symmetric distribution of the 21-nt sRNA matches (Figure 5H). In this element, the perfectly paired regions of the TIRs are the main target of the sRNA. In contrast, the different *MITE Thalos *42j2-9 has two nucleotide changes in the 3'-TIR (Figure 5I) that greatly reduce the sRNA counts in this region. In *Thalos *BQ620108-1 a single nucleotide change in the 5'-TIR also reduces sRNA counts (Figure 5J).

## Discussion

### Many wheat sRNAs target transposable elements

The profile of sRNAs perfectly matching the five wheat genomic regions analyzed here suggests that many wheat sRNAs are produced from TEs. These results are in general agreement with whole-genome studies of other plant species, in spite of the fact that the five analyzed regions comprise only 7.5 × 10 ^-5 ^% of the wheat genome (~17 Gb) and were selected from gene rich regions providing only a partial view of the wheat genome. In *Arabidopsis thaliana *most of the sRNAs correspond to transposons and repeats, and the highest densities of 24-nt sRNA-matching regions were detected in the centromeric and pericentromeric regions, where DNA transposons and retrotransposons are highly abundant [22,64,82]. In rice, large numbers of sRNAs originate from retrotransposon or transposon-related sequences [83]. Our analysis is limited to gene-rich regions of the wheat genome and we cannot rule out that a different profile of perfectly matching sRNAs is present in gene-poor regions, which represent most of the wheat genome.

In spite of the preponderance of sRNAs matching TEs in the five genomic regions analyzed here, a search of the TREP database perfectly matched only 6% of the sRNAs in our consolidated wheat sRNA database. Many of the non-matching sRNAs likely originate from intergenic regions [64], pseudogenes [84], or gene coding loci [64], but many may match undiscovered TE families or members of known families that have sufficiently diverged from their representatives in TREP. Most of the wheat genomic regions currently deposited in GenBank are the result of map-based positional cloning efforts and, therefore, are focused on gene-rich regions, whereas a large proportion of TEs are present in gene-poor regions [85,86]. Although this bias is likely reflected in the TEs currently present in TREP, the relative abundance of the different superfamilies in TREP was confirmed by the analysis of 1X shotgun sequencing of the complete hexaploid wheat genome (http://www.cerealsdb.uk.net/search_reads.htm; K.J. Edwards, personal communication). In addition, by considering only perfect matches, our analysis may not include an adequate sampling of the diversity of TE sequences within families and may underestimate the sRNA targets, since some sRNAs can be also effective against imperfectly matched targets [87,88]. The wheat TEs with the highest number of perfectly matching sRNAs include the class I *Copia *and *Gypsy *retrotransposons and the class II MITEs, which are also likely the most abundant TEs in the wheat genome (Table 2). A similar situation is observed in barley where the sRNAs matching the *Copia*, *Gypsy *and *CACTA *TEs account for 83% of the perfect matching barley sRNAs and together represent more than 50% of the matches to the random sequencing of 1% of the haploid barley genome [86]. However, the correspondence between TE abundance and number of perfect matches to the sRNAs is not perfect. In the sampled genomes of both wheat and barley, *Gypsy *TEs (38% in wheat and 48% in barley) are more abundant than *Copia *TEs (19% in wheat and 27% in barley), but the number of sRNAs with perfect matches is higher in *Copia *(47% in wheat and 53% in barley) than in *Gypsy *(18% in wheat and 15% in barley) TEs. Based on the different estimates of TE abundance it can be estimated that 6- to 8-fold more sRNAs match *Copia *than *Gypsy *TEs. Their relative levels of expression cannot explain this difference since the abundance of TEs from these two classes in the EST collections seems to be proportional to the number of copies (Table 2). Since most of the sRNAs are targeted to the LTRs we speculated that longer LTRs in the *Copia *relative to the *Gypsy *TEs could provide an explanation. However, this was not the case since, although very variable in size (Additional File 1 Figure S2), average LTR lengths were longer in *Gypsy *(2,045 bp) than *Copia *(1,110 bp) TEs. In summary, the excess of sRNAs matching the *Copia *TEs remains to be explained.

### Different classes of wheat TEs are targeted by different classes of sRNAs

The 24-nt sRNA are involved in RNA-dependent DNA methylation and heterochromatin maintenance and thus suppress transcription from DNA, whereas the 21-nt sRNAs regulate the half life and translation of related mRNAs [49,51,89]. It is interesting that most of the TE-matching sRNAs in wheat belong to the 24-nt group, whereas *MITEs *are preferentially matched by 21-nt sRNAs.

These results suggest that the activity of *MITE *TEs is regulated primarily after transcription, while the activity of all the other TE families is regulated by repression of transcription. In addition, the restricted targeting of the 21-nt sRNAs observed within the TIR regions of *MITEs *is more similar to the pattern of regulatory sRNAs in *A. thaliana *and rice than to the more dispersed distribution of 24-nt sRNAs [64,83]. Unlike other TEs, *MITEs *often occur in 5' or 3' UTRs of genes and sometimes even integrate in the coding sequences [13]. In consequence, *MITEs *are often expressed as read-through transcripts [46]. *MITEs *are flanked by short TIRs joined by little or no spacer DNA [46] that when expressed as RNA form a highly stable hairpin loop, which can then be recognized and processed by the RNA interference enzymes into mature 21-nt RNA [46].

The preponderance of 21-nt *MITE *sRNAs in polyploid wheat contrasts with the preponderance of 24-nt *MITE *sRNAs reported in diploid plant species [90]. Wheat is a recent polyploid with a high level of gene redundancy, and therefore, has a high tolerance to genic mutations [67], which may allow the accumulation of *MITEs *in genic regions. Besides introducing a target site for silencing, the insertion of a TE in a coding region may introduce an alternative polyadenylation site when located in the 3' UTR, affect mRNA stability and translation initiation, or interfere with the normal splicing pattern and perturb the functionality of the resulting protein [46].

Retrotransposons and DNA transposons other than *MITEs *were preferentially associated with 24-nt sRNAs. As in the case of *MITEs*, in DNA transposons such as the *CACTA *elements, read-though transcription and intramolecular pairing of inverted repeats may underlie the generation of dsRNA and consequently of sRNAs [19]. The higher sRNA counts matching the terminal repeats, including the subterminal inverted repeats may reflect not only the higher degree of conservation of these regions [81], but also a mechanism of sRNA generation based on the formation of terminal dsRNA loops.

In the case of the class I TEs, dsRNA can be generated by RNA-dependent RNA polymerase (RdRP) [91] or by intermolecular pairing of antiparallel transcripts [19]. For example, the bidirectional transcription of retrotransposons leads to the generation of sRNAs and to silencing in human cells [92] and in *Drosophila melanogaster *[93].

### sRNAs match specific areas of the repetitive elements

The sRNAs matching wheat *Copia *and *Gypsy *TEs were concentrated in the LTRs (Figure 5), a pattern also observed in maize [94]. LTRs do not encode for known proteins, but contain the promoters and terminators required for the transcription of the retroelement and are partially transcribed [95].

The significantly larger proportion of sRNAs matching LTRs may simply reflect the higher abundance of LTRs relative to the internal domain region in the genome. In addition to the natural duplication of the LTR at both ends of the TE, the inter-LTR region is eliminated in a large proportion of TEs resulting in solo-LTRs. For example, barley contains an average of 15 solo-LTRs per internal domain [96]. The significantly larger proportion of sRNAs matching LTRs might also indicate a higher chance of antiparallel pairing of these repetitive regions, or that LTRs are targeted by RdRP, or that the plant-specific DNA dependent RNA polymerase IVa (PolIVa) at the DNA level uses LTRs as template to generate sRNAs [97-100].

### Regions targeted by sRNAs have higher rates of mutation

The presence of abundant sRNAs matching TEs suggests that epigenetic mechanisms are involved in the silencing of their expression and motility in wheat. The sRNA-metabolic pathway guides both the *de novo *methyltransferases to initiate DNA methylation at direct repeats [101] and the maintenance methyltransferases responsible for remethylation and the maintenance of the transgenerational stability of the heavily methylated repetitive elements [102]. In *A. thaliana*, 24-nt sRNAs are generated by the DICER-LIKE 3 protein and, when loaded in one of the ten argonaute proteins, AGO4, target DNA methylation [50].

In plants, sRNAs induce methylation of not only CG dinucleotides, which are the primary sites of methylation in mammals, but also cytosines in the CHG and CHH sequence contexts [79]. Our estimations of DNA methylation based on transition rates parallel the higher methylation of cytosines in the CG and CHG contexts compared to CHH observed in Arabidopsis [79]. In wheat, TEs are preferentially methylated compared to introns and untranslated genic regions in agreement with studies in *A. thaliana*, maize, and primates [77,79,103]. The importance of DNA methylation is evidenced by the dramatic increase in TE transcription observed in methylation-deficient mutants of *A. thaliana *[54,79,104]. Methylated cytosine may affect TE expression either directly by interfering with the proper binding of proteins involved in transposition or indirectly by recruiting methylcytosine binding proteins that in turn associate with complexes containing co-repressors and histone deacetylases that modify chromatin structure [105].

In addition to the rapid and reversible TE repression, DNA methylation can irreversibly inactivate TEs by increasing the mutation frequency of the methylated sites. Methylated cytosines spontaneously deaminate to form thymine at a faster rate than non-methylated cytosines in the same sequence context [78]. Based on the estimated divergence time between *T. monococcum *and *T. turgidum *of 1.1 MYA [67] and an estimated nucleotide substitution rate in the introns and untranslated regions of 5.5 x 10 ^-9 ^nt ^-1 ^year ^-1 ^[106], we estimated that the substitution rate was 1.6 x 10 ^-8 ^nt ^-1 ^year ^-1 ^in the TEs (1,643 nucleotides considered, including 431 transitions and 156 transversions), which is about three times faster than the substitution rate in the untranslated genic regions. This estimate is almost identical to the one obtained previously for a different wheat genomic region [107]. This higher substitution rate in TEs is paralleled by a significantly higher transition rates but not any difference in the rate of transversions. Thus cytosine methylation and subsequent transition account for the faster substitution rate observed in the TEs.

The sequence erosion initiated by DNA methylation may account for the smaller number of sRNAs derived from older TEs relative to the number from more recently inserted TEs (Figure 1). The higher mutation rate in methylated TEs together with high rates of deletions in the intergenic regions may contribute to the permanent inactivation of TEs, [67,81].

## Conclusions

Our study provides a first exploration of the wheat epigenome and its close connection with the TEs that compose the vast majority of the wheat genome. Our findings suggest that sRNA-directed transcriptional and post-trascriptional silencing suppress TE activity in the wheat genome. DNA methylation and the consequent increase in the mutation rate at the methylated sites may silence TEs more permanently.

## Methods

### sRNA database construction

For the TAE4 library, transgenic plants for the *TaNAM *RNAi construct [68] were grown under long-days (16 h light 8 h dark). The experiment was originally performed to characterize the production of sRNAs from the NAM-RNAi transgene, but it then expanded beyond the original objective. At anthesis, spikes were labelled and after 12 days, flag-leaves samples from four plants were pooled and used for RNA extraction. Total RNA was prepared using the TRIZOL reagent (Invitrogen, Carlsbad, CA, USA) and its integrity was evaluated by gel electrophoresis. The TAE4 sRNA library was prepared using Illumina Small RNA Library Sample Prep protocol version 1.5. Total RNA (10 ug) was used as input material. The 3' adaptor (Illumina, San Diego, CA, USA) was ligated to RNAs. The v1.5 3' adapter predominantly ligates to microRNAs and other small RNAs that have a 3' hydroxyl group. The 3' ligation products were then ligated to the 5' small RNA adapter (Illumina). The ligation products were reverse transcribed followed by PCR amplification. The amplification products from 18 - 30 nt long RNAs were excised from a Novex 6% TBE PAGE gel (Invitrogen). The purified DNA fragments were submitted for 45 cycles of sequencing on the Illumina Genome Analyzer. The resulting sequencing reads were filtered for quality, and then trimmed to remove the sequence of 3' adapters. Filtering and trimming scripts are available from http://code.google.com/p/atgc-illumina/. Only the high quality reads with detectable 3' adapter were used for the analysis. The sequence data were deposited in the National Center for Biotechnology Information's Gene Expression Omnibus (GEO; [108]) and are accessible through GEO (accession n. GSM548032; http://www.ncbi.nlm.nih.gov/geo/query/acc.cgi?acc= GSM548032). TAE1, TAE2, and TAE3 sRNA libraries were obtained from the comparative sequencing project described on http://smallrna.udel.edu, where details on the libraries are available [94]. TAE1, TAE2, TAE3, and TAE4 sRNAs libraries were integrated in a single database using MySQL5.1 (MySQL AB; http://www.mysql.com/). For the analysis of barley sRNAs we used the barley sRNA libraries HVU1, HVU2, and HVU3, obtained from the comparative sequencing project http://smallrna.udel.edu.

The program "dbmanager.py" written in Python 2.6 for the database setup is provided in Additional File 1 Text S1.

### Analysis of perfect matching sRNAs

A computer program,"srna_seeker.py" (Additional File 4) written in Python 2.6 was used to count the perfectly matching sRNAs to both BAC sequences and TEs. The program consisted in a scrolling window with frame size between 18 and 33 nt that scans through the entire query sequence with a 1 nt increment after each read ("scanner.py", Additional File 3). When the scanner finds a region of the query sequence that exactly matches a sRNA in the database, "srna_seeker.py" returns the matching sRNA sequence, the length of the sequence (18 - 33 nt), the coordinate within the queried region, the library of origin, and the total counts in the sRNA database.

### Estimation of cytosine methylation

A computer program, "Cmet_scan.py" (Additional File 5) written in Python 2.5, was used to estimate the frequency of mutations in potentially methylated sites (CG, CHG and CHH). This measure is used as a proxy to infer the methylation state of these regions, since methylated C mutates three-times more frequently than unmethylated C (see main text). The program was used to compare repetitive and low copy number regions in orthologous genomic sequences of *T. monococcum *and *T. turgidum *and 5' and 3' long tandem repeats (LTRs) flanking Copia and Gypsy retroelements. Orthologous regions were aligned with ClustalW which generates outputs with "-" characters in the indels. The aligned FASTA file sequences were used as input for Cmet_scan.py. All Cs or Gs in both genomes in any of the CG, CHG, and CHH nucleotide contexts were considered as potentially methylated sites (PMS). G sites in one DNA strand are C in the opposite strand, and can be methylated. Therefore, both C and G sites were counted as potentially methylated sites. Bases paired with "-" (indels) were excluded from all calculations. C and G sites were classified as CG, CHG, or CHH. Presence of a CG or CHG in one genome was considered sufficient to count both the C and the G as potentially methylated. Detailed examples are included in the program file (Additional File 5). Cmet_scan.py counts all nucleotide substitutions, either transitions or transversions, and returns separately the percentage of transitions and transversions from CG, CHG, and CHH, for the complete sequence and for the total PMS.

## Authors' contributions

DC, LSV, MM, and AD carried out the experiments; DC, LSV, MM, and JD performed data analysis; DC, AS, MD, and JD developed the computer programs; DC, LSV, JD performed the statistical analyses; JD, RWM, and MM helped with the interpretation of the results; DC drafted the manuscript; JD and RWM were involved in improving the manuscript. All the authors approved the final version of the manuscript.

## Supplementary Material

Additional file 1 **Figure S1 - sRNA counts in TE families**. Box plots represent the distribution of the total counts of sRNA perfectly matching wheat TEs of each family in the seven major superfamilies deposited in the TREP database. Numbers above the whiskers represent the number of TREP elements within each superfamily considered. **Figure S2 **- LTR length in *Copia *and *Gypsy *TEs. Box plots represent the distribution of LTR lengths in the *Copia *and *Gypsy *elements deposited in the TREP database. **Table S1 - **Summary of sRNA libraries. **Table S2 - **Distribution of sRNA counts in the EU835198 genomic region (*T. turgidum*). **Table S3 - **Distribution of sRNA counts in the DQ871219 genomic region (*T. turgidum*). **Table S4 - **Distribution of sRNA counts in the EF540321 genomic region (*T. turgidum*). **Table S5 - **Distribution of sRNA counts in the EF567062 genomic region (*T. aestivum*). **Table S6 - **Distribution of sRNA counts in the DQ537335 genomic region (*T. aestivum*). **Table S7 - **sRNA counts in the different TE families. **Table S8 - **Estimates of LTR age of insertion and cytosine methylation in the CG, CHG, and CHH contexts. Click here for file

Additional file 2 **dbmanager.py **- python program for sRNA database setup. Click here for file

Additional file 3 **scanner.py - **python program for scrolling window analysis with frame size between 18 and 33 nt that scans through the entire query sequence with a 1 nt increment after each read. Click here for file

Additional file 4 **srna_seeker.py **- program for scanning nucleotide sequences for perfect matching sRNAs using "dbmanager.py" to access the sRNA database and "scanner.py" to analyze the query sequences. Click here for file

Additional file 5 **Cmet_scan.py **- python program for counting mutations in potentially methylated sites. Click here for file
