# Supplementary material for: Small RNAs, DNA methylation and transposable elements in wheat
Source: BMC Genomics. 2010 Jun 29;11:408. doi: 10.1186/1471-2164-11-408 (PMC2996936; doi:10.1186/1471-2164-11-408)
Supplement: Additional file 1 — Figure S1 - sRNA counts in TE families. Box plots represent the distribution of the total counts of sRNA perfectly matching wheat TEs of each family in the seven major superfamilies deposited in the TREP database. Numbers above the whiskers represent the number of TREP elements within each superfamily considered. Figure S2 - LTR length in Copia and Gypsy TEs. Box plots represent the distribution of LTR lengths in the Copia and Gypsy elements deposited in the TREP database. Table S1 - Summary of sRNA libraries. Table S2 - Distribution of sRNA counts in the EU835198 genomic region (T. turgidum). Table S3 - Distribution of sRNA counts in the DQ871219 genomic region (T. turgidum). Table S4 - Distribution of sRNA counts in the EF540321 genomic region (T. turgidum). Table S5 - Distribution of sRNA counts in the EF567062 genomic region (T. aestivum). Table S6 - Distribution of sRNA counts in the DQ537335 genomic region (T. aestivum). Table S7 - sRNA counts in the different TE families. Table S8 - Estimates of LTR age of insertion and cytosine methylation in the CG, CHG, and CHH contexts. [file 1471-2164-11-408-S1.PDF]

Additional file 1

Additional File 1 Figure S1 - sRNA counts in TE families. Box plots represent the distribution of the total counts of sRNA perfectly matching wheat TEs of each family in the seven major superfamilies deposited in the TREP database. Numbers above the whiskers represent the number of TREP elements within each superfamily considered.

Additional File 1 Figure S2 - LTR length in *Copia* and *Gypsy* TEs. Box plots represent the distribution of LTR lengths in the *Copia* and *Gypsy* elements deposited in the TREP database.

Additional File 1 Table S1 - Summary of sRNA libraries.

Additional File 1 Table S2 - Distribution of sRNA counts in the EU835198 genomic region (*T. turgidum*).

Additional File 1 Table S3 - Distribution of sRNA counts in the DQ871219 genomic region (*T. turgidum*).

Additional File 1 Table S4 - Distribution of sRNA counts in the EF540321 genomic region (*T. turgidum*).

Additional File 1 Table S5 - Distribution of sRNA counts in the EF567062 genomic region (*T. aestivum*).

Additional File 1 Table S6 - Distribution of sRNA counts in the DQ537335 genomic region (*T. aestivum*).

Additional File 1 Table S7 - sRNA counts in the different TE families.

Additional File 1 Table S8 - Estimates of LTR age of insertion and cytosine methylation in the CG, CHG, and CHH contexts.

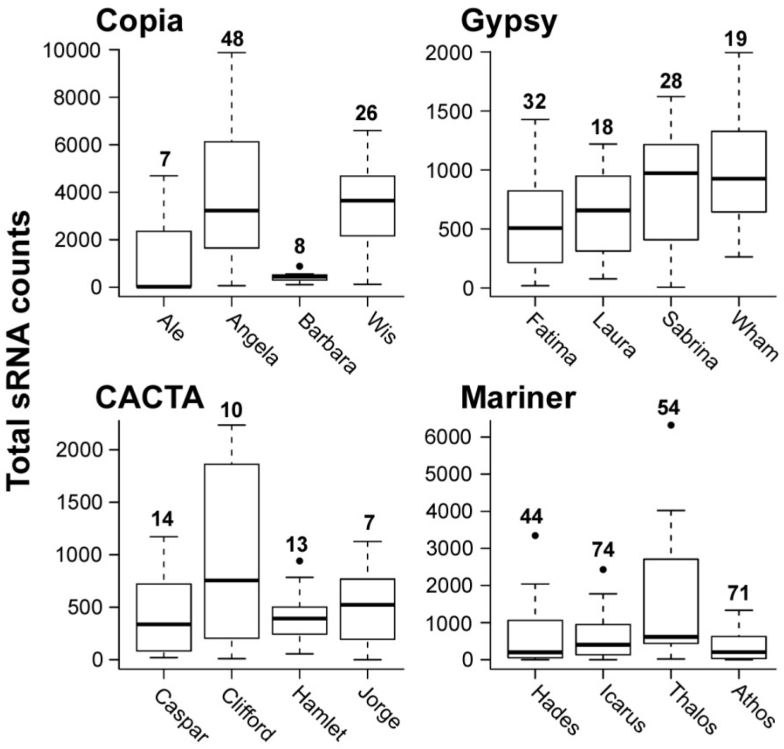

Supplemental Figure S1 – sRNA counts in TE families. Box plots represent the distribution of the total counts of sRNA perfectly matching wheat TEs of each family in the seven major superfamilies deposited in the TREP database. Numbers above the whiskers represent the number of TREP elements within each superfamily considered.

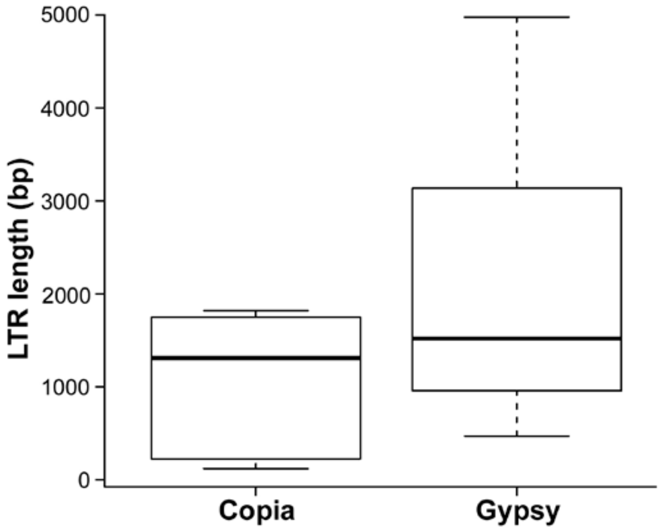

Supplemental Figure S2 – LTR length in *Copia* and *Gypsy* TEs. Box plots represent the distribution of LTR lengths in the *Copia* and *Gypsy* elements deposited in the TREP database.

Suplemental Table S1 - Summary of sRNA libraries

Library	Sequences	Distinct Sequences	21 nt (%)	24 nt (%)
TAE1	3,570,129	924,133	24.4	9.8
TAE2	2,916,955	1,661,668	12.3	54.2
TAE3	2,968,383	1,153,689	10.6	27.7
TAE4	1,074,691	331,340	29.7	25
Total	10,530,158	3,755,852	17.7	28.7

Supplemental Table S2 - Distribution of sRNA counts in the EU835198 genomic region (*T. turgidum*)

Annotation	Class	Order	Super Family	Family	5' coordinate	3' coordinate	Length	sRNA Counts	sRNA Counts/base	21 nt	24 nt	%21 nt	%24 nt
Wis_391M13_1	I	LTR retrotransposons	Copia	Wis	1	1272	1272	1584	1.25	205	706	12.94	44.57
Jeli_391M13_1	I	LTR retrotransposons	Gypsy	Jeli	8726	52867	12587	296	0.02	74	119	25.00	40.20
Egug_391M13_1	I	LTR retrotransposons	Gypsy	Egug	16279	30471	5409	385	0.12	37	240	9.61	62.34
Wis_391M13_2	I	LTR retrotransposons	Copia	Wis	17549	26332	8784	3051	0.35	289	1819	9.47	59.62
Wis_391M13_3	I	LTR retrotransposons	Copia	Wis	32749	41428	8680	3451	0.40	276	2320	8.00	67.23
Wis_391M13_4	I	LTR retrotransposons	Copia	Wis	42995	51668	8674	3542	0.41	302	2059	8.53	58.13
Hades_391M13_1	II	TIR transposons	Mariner	MITE Stowaway	54221	54278	58	96	1.66	74	1	77.08	1.04
Athos_391M13_1	II	TIR transposons	Mariner	MITE Stowaway	56579	56661	83	312	3.76	283	9	90.71	2.88
Leojyg_391M13_1	I	LTR retrotransposons	Copia	Leojyg	58014	63159	5146	110	0.02	4	85	3.64	77.27
CACTA-like (rice)	II	TIR transposons	CACTA		64508	65047	540	1	0.00	-	-	-	-
Jeli_391M13_2	I	LTR retrotransposons	Gypsy	Jeli	65048	95387	13018	513	0.04	134	196	26.12	38.21
Wis_391M13_5	I	LTR retrotransposons	Copia	Wis	71748	80371	8624	4753	0.55	506	2299	10.65	48.37
Wis_391M13_6	I	LTR retrotransposons	Copia	Wis	83139	91831	8693	4909	0.56	573	2522	11.67	51.38
CACTA-like (rice)	II	TIR transposons	CACTA		95388	96619	1232	7	0.01	0	5	0.00	71.43
IBR1	GENE				99666	100232	567	0	0.00	-	-	-	-
Sabrina_391M13_1	I	LTR retrotransposons	Gypsy	Sabrina	106117	107681	1565	24	0.02	6	12	25.00	50.00
Ale_391M13_1	I	LTR retrotransposons	Copia	Ale	112583	117931	5349	1100	0.21	72	829	6.55	75.36
Athos_391M13_4	II	TIR transposons	Mariner	MITE Stowaway	120586	120666	81	676	8.35	582	24	86.09	3.55
Angela_391M13_1	I	LTR retrotransposons	Copia	Angela	121686	135280	4858	4279	1.68	550	2010	12.85	46.97
Wis_391M13_7	I	LTR retrotransposons	Copia	Wis	124937	133673	8737	4735	0.54	404	2803	8.53	59.20
Sukkula_258C22-1 like	I	LTR retrotransposons	Gypsy	Sukkula	135365	136265	901	5	0.01	1	3	20.00	60.00
Enac_426K20-1 like	II	TIR transposons	CACTA		136535	136834	300	139	0.46	18	95	12.95	68.35
WKS1	GENE				138832	146118	7287	0	0.00	-	-	-	-
Hades_391M13_2	II	TIR transposons	Mariner	MITE Stowaway	140745	140802	58	4	0.07	3	0	75.00	0.00
Oleus_391M13_1	II	TIR transposons	Mariner	MITE Stowaway	143573	143731	159	1	0.01	-	-	-	-
Thalos_391M13_1	II	TIR transposons	Mariner	MITE Stowaway	144705	144865	161	546	3.39	72	292	13.19	53.48
Democles_391M13_1	II	TIR transposons	Mariner	MITE Stowaway	153180	153340	161	2175	13.51	1915	140	88.05	6.44
Sabrina_391M13_2	I	LTR retrotransposons	Gypsy	Sabrina	159383	161612	2230	478	0.21	37	331	7.74	69.25
WKS2	GENE				165749	173407	7659	0	0.00	-	-	-	-
Karin_391M13_1	I	Non LTR retrotransposons	LINE	Karin	169514	172687	3174	475	0.15	59	341	12.42	71.79
Fatima_1144M20_1	I	LTR retrotransposons	Gypsy	Fatima	175522	245717	9210	206	0.02	23	115	11.17	55.83
Bare_1144M20_1	I	LTR retrotransposons	Copia	Bare	176366	197150	8475	5343	1.26	592	2880	11.08	53.90
Jeli_1144M20_1	I	LTR retrotransposons	Gypsy	Jeli	180702	193011	12310	7654	0.62	2046	2289	26.73	29.91
Angela_1144M20_1	I	LTR retrotransposons	Copia	Angela	199288	239478	8531	6161	0.72	620	3648	10.06	59.21
Erika_1144M20_1	I	LTR retrotransposons	Gypsy	Erika	201343	219559	5641	799	0.14	238	206	29.79	25.78
CACTA_1144M20_1	II	TIR transposons	CACTA		201435	206025	4591	61	0.01	5	21	8.20	34.43
Angela_1144M20_2	I	LTR retrotransposons	Copia	Angela	206026	214010	7985	4212	0.53	524	2377	12.44	56.43
Erika_1144M20_2	I	LTR retrotransposons	Gypsy	Erika	220573	234005	4991	516	0.10	146	171	28.29	33.14
Angela_1144M20_3	I	LTR retrotransposons	Copia	Angela	223200	231641	8442	5958	0.71	837	2742	14.05	46.02
Leojyg_1144M20_1	I	LTR retrotransposons	Copia	Leojyg	258136	263283	5148	109	0.02	4	86	3.67	78.90
Athos_1144M20_1	II	TIR transposons	Mariner	MITE Stowaway	264332	264415	84	2	0.02	1	1	50.00	50.00
CACTA-like (rice)	II	TIR transposons	CACTA		264632	265171	540	0	0.00	-	-	-	-
Jeli_1144M20_2	I	LTR retrotransposons	Gypsy	Jeli	265172	278183	13012	525	0.04	139	213	26.48	40.57
CACTA-like (rice)	II	TIR transposons	CACTA		278184	279415	1232	5	0.00	0	3	0.00	60.00
IBR2	GENE				280707	281273	567	0	0.00	-	-	-	-
Predicted Pectate Lyase	GENE				291816	294033	2218	8	0.00	3	2	37.50	25.00
Fatima_1144M20_2	I	LTR retrotransposons	Gypsy	Fatima	294273	307419	8161	197	0.02	27	108	13.71	54.82
Erika_1144M20_3	I	LTR retrotransposons	Gypsy	Fatima	294971	299956	4986	1279	0.26	353	366	27.60	28.62
Jeli_1144M20_3	I	LTR retrotransposons	Gypsy	Fatima	307420	314057	6638	134	0.02	19	79	14.18	58.96

Supplemental Table S3 – Distribution of sRNA counts in the DQ871219 genomic region (*T. turgidum*)

Annotation	Class	Order	Super Family	Family	5' coordinate	3' coordinate	Length	sRNA Counts	sRNA Counts/base	21 nt	24 nt	%21 nt	%24 nt
Barbara_609E6-like	I	LTR retrotransposon	Copia	Barbara	2528	3597	1070	20	0.02	3	14	15.00	70.00
CACTA_916017_1	II	TIR transposon	CACTA		3598	53589	11570	946	0.08	43	492	4.55	52.01
Sabrina_916017_1	I	LTR retrotransposon	Gypsy	Sabrina	8918	29273	1570	335	0.21	51	186	15.22	55.52
Fatima_916017_1	I	LTR retrotransposon	Gypsy	Fatima	9746	28471	9949	711	0.07	63	440	8.86	61.88
Wis_916017_1	I	LTR retrotransposon	Copia	Wis	17700	26476	8777	3019	0.34	232	2005	7.68	66.41
Sabrina_916017_1 Solo LTR	I	LTR retrotransposon	Gypsy	Sabrina	54129	54356	228	6	0.03	-	-	-	-
Sabrina_916017_2 Solo LTR	I	LTR retrotransposon	Gypsy	Sabrina	54357	57338	2891	601	0.21	56	386	9.32	64.23
Athos_916017_1	II	TIR transposon	Mariner	MITE Stowaway	55954	56034	81	815	10.06	702	26	86.13	3.19
Wis_916017_2	I	LTR retrotransposon	Copia	Wis	58319	65549	7231	3503	0.48	242	1906	6.91	54.41
Barbara_916017_2	I	LTR retrotransposon	Copia	Barbara	66467	67789	1323	2	0.00	-	-	-	-
NAM-B1		GENE			75858	80616	4759	0 (4105)*	0 (0.86)*	3616	259	88.09	6.31
Hades_916017_1	II	TIR transposon	Mariner	MITE Stowaway	88130	88211	82	280	3.41	158	36	56.43	12.86
Inav_AY013246-1 like	I	LTR retrotransposon	Copia	Inav	88371	89906	1536	2	0.00	0	2	0.00	100.00
Wilma_916017_1 like Solo LTR	I	LTR retrotransposon	Gypsy	Wilma	90084	90915	832	9	0.01	-	-	-	-
Karin_AF459088-1 like	I	Non LTR retrotransposon	LINE	Karin	93688	96796	3109	0	0.00	-	-	-	-
Egug_916017_1	I	LTR retrotransposon	Gypsy	Egug	103552	111127	7483	702	0.09	44	474	6.27	67.52
Hades_916017_2	II	TIR transposon	Mariner	MITE Stowaway	106896	106988	93	341	3.67	282	15	82.70	4.40
Karin_294D11-2 like	I	Non LTR retrotransposon	LINE	Karin	112919	115424	2506	0	0.00	-	-	-	-
CACTA_409D13_1	II	TIR transposon	CACTA		115428	186046	3605	112	0.03	16	70	14.29	62.50
Jeli_409D13_1	I	LTR retrotransposon	Gypsy	Jeli	115476	132068	13078	519	0.04	114	249	21.97	47.98
Sabrina_409D13_1	I	LTR retrotransposon	Gypsy	Sabrina	122230	131091	8862	1022	0.12	110	592	10.76	57.93
CACTA_409D13_2	II	TIR transposon	CACTA		132069	177142	10831	1031	0.10	73	506	7.08	49.08
Fatima_409D13_1	I	LTR retrotransposon	Gypsy	Fatima	134471	144856	10386	860	0.08	79	545	9.19	63.37
Polyphemus_409D13_1	II	TIR transposon	Mariner	MITE Stowaway	151081	151315	235	34	0.14	8	18	23.53	52.94
Eos_409D13_1	II	TIR transposon	Mariner	MITE Stowaway	151374	151718	345	506	1.47	63	270	12.45	53.36
Thalos_409D13_1	II	TIR transposon	Mariner	MITE Stowaway	152090	152246	157	2828	18.01	2426	181	85.79	6.40
Thalos_409D13_2	II	TIR transposon	Mariner	MITE Stowaway	154775	154941	167	18	0.11	7	4	38.89	22.22
Sabrina_409D13_2	I	LTR retrotransposon	Gypsy	Sabrina	157203	165012	7810	814	0.10	88	510	10.81	62.65
Thalos_409D13_3	II	TIR transposon	Mariner	MITE Stowaway	172856	173016	161	3340	20.75	2829	230	84.70	6.89
Karin_294D11-2 like	I	Non LTR retrotransposon	LINE	Karin	186047	187052	1006	2	0.00	0	1	0.00	50.00
putative rhamnogalacturonate lyase		GENE			187999	190299	2301	0	0.00	-	-	-	-
Angela_409D13_1	I	LTR retrotransposon	Copia	Angela	193994	214109	8606	5808	0.67	804	2748	13.84	47.31
Wis_409D13_1	I	LTR retrotransposon	Copia	Wis	196386	204902	8517	4724	0.55	501	2442	10.61	51.69
Wis_409D13_2	I	LTR retrotransposon	Copia	Wis	204903	206148	1246	159	0.13	14	104	8.81	65.41
Wis_409D13_3 solo LTR	I	LTR retrotransposon	Copia	Wis	208420	210162	1743	996	0.57	68	747	6.83	75.00
Isabelle_AF326781-1 like	I	Non LTR retrotransposon	LINE	Isabelle	214500	217035	2536	0	0.00	-	-	-	-
Deimos_409D13_1	I	TIR transposon	Mutator	Deimos	218346	218810	465	66	0.14	2	49	3.03	74.24
Ale_AF459088-1 like	I	LTR retrotransposon	Copia	Ale	221961	226500	4540	35	0.01	6	20	17.14	57.14
predicted leucine rich repeats		GENE			226966	229839	2874	55	0.02	2	48	3.64	87.27
MITE_409D13_1	II	TIR transposon	Mariner	MITE Stowaway	235886	235966	81	9	0.11	7	0	77.78	0.00
Pan_409D13_1	II	TIR transposon	Mariner	MITE Stowaway	236139	236261	123	3199	26.01	1839	928	57.49	29.01
RNA rec motif RRM-cont protein		GENE			237280	239897	2618	3	0.00	-	-	-	-
putative calcium exchanger		GENE			240808	243579	2772	1	0.00	-	-	-	-

* Numbers in parenthesis represent the perfectly matching sRNAs of the TAE4 sRNA library

Supplemental Table S4 - Distribution of sRNA counts in the EF540321 genomic region (*T. turgidum*)

Annotation	Class	Order	Super Family	Family	5' coordinate	3' coordinate	Length	Counts	Counts/base	21	24	%21	24%
Erika_326E2_1	I	LTR retrotransposons	Gypsy	Erika	1	4038	4038	1015	0.25	243	411	23.94	40.49
Latidu_326E2_1	I	LTR retrotransposons	Gypsy	Latidu	4039	4674	636	18	0.03	3	9	16.67	50.00
Angela_326E2_1	I	LTR retrotransposons	Copia	Angela	5692	7437	1746	336	0.19	47	171	13.99	50.89
Gerald_326E2_1 like	II	TIR transposon	Mutator	Gerald	7864	8188	325	9	0.03	8	1	88.89	11.11
PS decarboxylase	GENE				12872	13609	738	2	0.00	2	0	-	-
AAS58483.1 like	GENE				20043	22884	2842	1	0.00	0	0	-	-
Angela_326E_2	I	LTR retrotransposons	Copia	Angela	23816	26082	2267	358	0.16	47	192	13.13	53.63
Jorge_326E2_1	II	TIR transposon	CACTA	Jorge	26083	40603	4128	738	0.18	181	206	24.53	27.91
Caspar_326E_1	II	TIR transposon	CACTA	Caspar	26597	36992	10396	820	0.08	79	578	9.63	70.49
Stowaway_326E2_1	II	TIR transposon	Mariner	MITE Stowaway	48911	48991	81	119	1.47	65	21	54.62	17.65
Stowaway_326E2_2	II	TIR transposon	Mariner	MITE Stowaway	52837	52959	123	1304	10.60	838	105	64.26	8.05
Stowaway_326E2_3	II	TIR transposon	Mariner	MITE Stowaway	61679	61779	101	1	0.01	0	1	-	-
Wham_326E2_1	I	LTR retrotransposons	Copia	Athila	62370	70904	3825	465	0.12	50	280	60.22	12.16
Putative ribosomal protein	GENE				74052	75098	1047	7	0.01	0	5	0.00	71.43
Isacc_326E2_1	II	TIR transposon	CACTA	Isaac	81204	90240	9037	117	0.01	13	62	11.11	52.99
Fatima_326E_1	I	LTR retrotransposons	Gypsy	Fatima	93405	103410	10006	800	0.08	72	500	9.00	62.50
ABA98678.1 like	II	putative transposon (similar to ABA98678.1)			103822	105925	2104	15	0.01	1	10	6.67	66.67
Vincent_326E2_1	II	TIR transposon	CACTA	Vincent	111340	115562	4223	100	0.02	10	75	10.00	75.00
Paula_362E2_1	I	Non-LTR retrotransposons	LINE	Paula	129538	130192	655	5	0.01	0	4	0.00	80.00
Yvonne_326E2_1	I	Non-LTR retrotransposons	LINE	Yvonne	131671	133168	1498	4	0.00	1	2	25.00	50.00
CAE05840 like	II	TIR transposon	Mutator		137869	141144	3276	9	0.00	2	2	22.22	22.22
CAD40214.1 like	I	Retrotransposon similar to rice gag protein CAD40214.1			143662	145110	1449	0	0.00	0	0	-	-
Stowaway_354M17_1	II	TIR transposon	Mariner	MITE Stowaway	151982	152062	81	291	3.59	263	7	90.38	2.41
CCT transcription factor	GENE				152570	153780	1211	0	0.00	0	0	-	-
AAP44645.1 like	I	putative retrotransposon (similar to AAP44645.1)			160128	160367	240	6	0.03	1	4	16.67	66.67
ABA96203	I	putative retrotransposon (similar to ABA96203)			165646	166820	1175	0	0.00	0	0	-	-
ZCCT2	GENE				170022	171738	1717	0	0.00	0	0	-	-
Stasy_354M17_1	I	Non-LTR retrotransposons	LINE	Stasy	173000	175876	2877	897	0.31	119	569	13.27	63.43
Martin_354M17_1	I	LTR retrotransposons		Martin	176830	178800	1971	16	0.01	1	15	6.25	93.75
VRN2	GENE				186587	188456	1870	0	0.00	0	0	-	-
Stowaway_354M17_2	II	TIR transposon	Mariner	MITE Stowaway	191701	191858	158	9	0.06	1	2	11.11	22.22
Ikeros_354M17_1	I	LTR retrotransposons	Copia	Ikeros	195094	200440	5347	156	0.03	19	112	12.18	71.79
Angela_354M17_1	I	LTR retrotransposons	Copia	Angela	205798	214507	8710	6781	0.78	733	3581	10.81	52.81
SNF2P	GENE				215906	222892	6987	3	0.00	2	1	66.67	33.33
Ramona_354M17_1	I	Non-LTR retrotransposons	LINE	Ramona	228720	232623	3904	9	0.00	1	4	11.11	44.44
Stowaway_354M17_3	II'	TIR transposon	Mariner	MITE Stowaway	236826	236927	102	10	0.10	2	6	20.00	60.00
Caspar_354M17_1	II	TIR transposon	CACTA	Caspar	239679	241529	833	170	0.20	15	128	8.82	75.29
Laura_354M17_1	I	LTR retrotransposons	Gypsy	Laura	242756	254933	12178	1202	0.10	306	641	25.46	53.33
Caspar_354M17_2	II	TIR transposon	CACTA	Caspar	256075	288431	5587	263	0.05	20	188	7.60	71.48
Angela_354M17_2	I	LTR retrotransposons	Copia	Angela	257769	275025	17257	7943	0.46	701	4973	8.83	62.61
Wis_354M17_1	I	LTR retrotransposons	Copia	Wis	257823	266448	8626	4868	0.56	399	3327	8.20	68.34
Angela_354M17_3	I	LTR retrotransposons	Copia	Angela	278161	286697	8537	6161	0.72	718	3406	11.65	55.28
Angela_354M17_4	I	LTR retrotransposons	Copia	Angela	288941	291163	2223	3201	1.44	351	1632	10.97	50.98

Supplemental Table S5 - Distribution of sRNA counts in the EF567062 genomic region (*T. aestivum*)

Annotation	Class	Order	Super Family	Family	5' coordinate	3' coordinate	Length	sRNA Counts	sRNA Counts/base	21	24	%21	24%
Sabrina-3	I	LTR retrotransposons	Gypsy	Sabrina	1	1846	1846	411	0.22	49	264	11.92	64.23
Fatima-1	I	LTR retrotransposons	Gypsy	Fatima	1847	5192	3346	118	0.04	16	70	59.32	13.56
WHAM-1	I	LTR retrotransposons	Copia	Athila	5193	14010	8818	1459	0.17	254	625	42.84	17.41
Angela-2	I	LTR retrotransposons	Copia	Angela	14011	34823	5434	2129	0.39	289	868	13.57	40.77
Sabrina-1	I	LTR retrotransposons	Gypsy	Sabrina	15778	22531	6754	1373	0.20	295	852	62.05	21.49
Angela-1	I	LTR retrotransposons	Copia	Angela	24920	33544	8625	7795	0.90	949	3636	12.17	46.65
Gypsy-1	I	LTR retrotransposons	Gypsy		34823	34980	158	0	0.00	0	0	-	-
Xabor-1					38123	39583	1461	37	0.03	0	31	0.00	83.78
Xusag-1					50797	52131	1335	10	0.01	0	7	0.00	70.00
Thalos-1	II	TIR transposon	Mariner	MITE Stowaway	53100	53278	179	7	0.04	3	3	42.86	42.86
Hades-1	II	TIR transposon	Mariner	MITE Stowaway	54267	54362	96	20	0.21	7	5	35.00	25.00
Lr1	GENE				54812	58846	4035	10	0.00	4	2	40.00	20.00
Polyphemus-3	II	TIR transposon	Mariner	MITE Stowaway	62963	63203	241	93	0.39	63	17	67.74	18.28
Karin-1	I	Non LTR retrotransposons	LINE	Karin	65497	72719	7223	136	0.02	14	96	10.29	70.59
Ramona-1	I	Non LTR retrotransposons	LINE	Ramona	73597	76530	2934	11	0.00	2	9	18.18	81.82
Caspar-5	II	TIR transposon	CACTA	Caspar	80654	81512	859	136	0.16	13	93	9.56	68.38
Caspar-4	II	TIR transposon	CACTA	Caspar	83761	83894	134	20	0.15	2	15	10.00	75.00
Caspar-1	II	TIR transposon	CACTA	Caspar	85877	88862	2986	457	0.15	58	282	12.69	61.71
Caspar-2	II	TIR transposon	CACTA	Caspar	88863	89421	559	720	1.29	49	542	6.81	75.28
RGA-1	GENE				89801	93859	4059	4	0.00	2	1	50.00	25.00
Xenon-1	II	TIR transposon	Harbinger	Tourist MITE	98585	100566	1982	40	0.02	6	25	15.00	62.50
Caspar-3	II	TIR transposon	CACTA	Caspar	101221	102616	1396	911	0.65	81	640	8.89	70.25
Argo-1	II	TIR transposon	Mutator	Argo	107671	108655	985	25	0.03	9	9	36.00	36.00
ABG-1	GENE				109962	113199	3238	16	0.00	9	2	56.25	12.50
Latidu-1	I	LTR retrotransposons	Gypsy	Latidu	117533	119936	2404	661	0.27	119	313	18.00	47.35
Polyphemus-2	II	TIR transposon	Mariner	MITE Stowaway	120749	120987	239	32	0.13	16	9	50.00	28.13
P450	GENE				121693	124695	3003	0	0.00	0	0	-	-
Sabrina2	I	LTR retrotransposons	Gypsy	Sabrina	127912	131257	2196	239	0.11	22	167	9.21	69.87
TAT-1	II	TIR transposon	CACTA	TAT	128401	129550	1150	108	0.09	13	81	12.04	75.00
TAT-2	II	TIR transposon	CACTA	TAT	131302	135543	4242	42	0.01	2	31	4.76	73.81
Daniela-1	I	LTR retrotransposons	Gypsy	Daniela	135544	136981	1438	18	0.01	1	11	5.56	61.11

Supplemental Table S6 - Distribution of sRNA counts in the DQ537335 genomic region (*T. aestivum*)

Annotation	Class	Order	Super Family	Family	5' coordinate	3' coordinate	Length	sRNA Counts	sRNA Counts/base	21	24	%21	24%
Receptor Kinase 1	GENE				3811	7851	4041	0	0.00	0	0	-	-
MITE-1	II	TIR transposon	Mariner	MITE Stowaway	8336	8421	86	2463	28.64	1978	206	80.31	8.36
MITE-2	II	TIR transposon	Mariner	MITE Stowaway	8867	8956	90	1	0.01	0	0	-	-
WIS-1p	I	LTR retrotransposons	Copia	Wis	12795	43579	8939	3425	0.38	346	1871	10.10	54.63
Angela-1p	I	LTR retrotransposons	Copia	Angela	21379	43224	8714	9370	1.08	789	5796	8.42	61.86
Erika-1	I	LTR retrotransposons	Gypsy	Erika	28801	41932	13132	978	0.07	401	534	41.00	54.60
MITE-3	II	TIR transposon	Mariner	MITE Stowaway	46074	46170	97	298	3.07	241	7	80.87	2.35
MITE-4	II	TIR transposon	Mariner	MITE Stowaway	46233	46493	261	117	0.45	23	83	19.66	70.94
MITE-5	II	TIR transposon	Mariner	MITE Stowaway	47123	47412	290	15	0.05	1	12	6.67	80.00
Ijeij-1	II	TIR transposon	CACTA		47573	54836	7264	130	0.02	47	19	36.15	14.62
MITE-6	II	TIR transposon	Mariner	MITE Stowaway	54887	54971	85	911	10.72	745	20	81.78	2.20
Madil-1p	I	LTR retrotransposons	Gypsy	Laura	55639	58710	3072	184	0.06	35	91	19.02	49.46
Boba-1	I	LTR retrotransposons	Copia	Boba	59781	73062	5409	10	0.00	2	8	20.00	80.00
WIS-2p	I	LTR retrotransposons	Copia	Wis	62395	70808	8414	4196	0.50	389	2456	9.27	58.53
MITE-7	II	TIR transposon	Mariner	MITE Stowaway	74637	74800	164	256	1.56	202	25	78.91	9.77
Globulin 1	GENE				76510	77199	690	11	0.02	2	7	18.18	63.64
MITE-8	II	TIR transposon	Mariner	MITE Stowaway	79626	79721	96	1872	19.50	1509	230	80.61	12.29
Y-type HMW glutenin	GENE				81033	82370	1338	6	0.00	0	6	0.00	100.00
Pivu-1	I	LTR retrotransposons	Gypsy	Sabrina	84297	230216	24201	1218	0.05	144	773	11.82	63.46
Latidu-1p	I	LTR retrotransposons	Gypsy	Latidu	90648	94776	4129	181	0.04	36	72	19.89	39.78
Wilma-2	I	LTR retrotransposons	Copia	Athila	97398	99250	1853	255	0.14	19	141	7.45	55.29
Hawi-1s	I	LTR retrotransposons	Gypsy	Hawi	99251	101903	2653	348	0.13	78	151	22.41	43.39
Wham-1p	I	LTR retrotransposons	Copia	Athila	101904	103163	1260	196	0.16	16	122	8.16	62.24
Wham-2p	I	LTR retrotransposons	Copia	Athila	103164	108221	5058	615	0.12	38	420	6.18	68.29
Wham-2p	I	LTR retrotransposons	Copia	Athila	103164	132995	5857	737	0.13	46	498	6.24	67.57
Jorge-1	II	TIR transposon	CACTA	Jorge	108222	132196	16002	980	0.06	67	485	6.84	49.49
Deimos-1	I	TIR transposon	Mutator	Deimos	115439	117622	2184	66	0.03	0	52	0.00	78.79
Gujog-1	I	LTR retrotransposons			118437	124226	5790	325	0.06	34	213	10.46	65.54
Wham-2p	I	LTR retrotransposons	Copia	Athila	132197	132995	799	122	0.15	8	78	6.56	63.93
Sabrina-1	I	LTR retrotransposons	Gypsy	Sabrina	147138	222274	6996	749	0.11	77	476	10.28	63.55
Lahuwi-1	I	LTR retrotransposons	Gypsy	Egug	153262	221401	11565	601	0.05	41	401	6.82	66.72
Nusif-1	I	LTR retrotransposons	Gypsy	Nusif	164148	220722	3870	39	0.01	9	22	23.08	56.41
Apiip-1	I	LTR retrotransposons	Gypsy	Danae	166645	194161	11236	34	0.00	6	16	17.65	47.06
Sabrina-2	I	LTR retrotransposons	Gypsy	Sabrina	167960	170008	2049	518	0.25	38	291	7.34	56.18
Fatima-1	I	LTR retrotransposons	Gypsy	Fatima	179085	184240	5156	946	0.18	102	555	10.78	58.67
Sabrina-3	I	LTR retrotransposons	Gypsy	Sabrina	197977	219349	8313	1155	0.14	108	724	9.35	62.68
Laura-1	I	LTR retrotransposons	Gypsy	Laura	200148	213207	13060	326	0.02	121	119	37.12	36.50
Sabrina-4	I	LTR retrotransposons	Gypsy	Sabrina	230332	259015	3797	98	0.03	19	51	19.39	52.04
Wilma-3s	I	LTR retrotransposons	Copia	Athila	231014	232530	1517	239	0.16	37	120	15.48	50.21
Fatima-2p	I	LTR retrotransposons	Gypsy	Fatima	234692	255846	9883	582	0.06	57	327	9.79	56.19
Hawi-2p	I	LTR retrotransposons	Gypsy	Hawi	238207	249478	11272	1290	0.11	322	541	24.96	41.94
Unknown-5s	I	LTR retrotransposons			255847	258061	2215	59	0.03	6	45	10.17	76.27
HMW glutenin gene	GENE				267842	270289	2448	14	0.01	0	13	0.00	92.86
protein kinase	GENE				270590	273168	2579	1	0.00	0	1	0.00	100.00
Angela-2p	I	LTR retrotransposons	Copia	Angela	277580	278385	806	654	0.81	104	378	15.90	57.80
Angela-3p	I	LTR retrotransposons	Copia	Angela	278386	290017	2013	1443	0.72	238	525	16.49	36.38
Fatima-3	I	LTR retrotransposons	Gypsy	Fatima	279541	289159	8087	106	0.01	13	54	12.26	50.94
Sabrina-5s	I	LTR retrotransposons	Gypsy	Sabrina	284169	285700	1532	696	0.45	86	378	12.36	54.31
Wham-3p	I	LTR retrotransposons	Copia	Athila	290018	290383	366	217	0.59	12	160	5.53	73.73
Nubude-1	I	LTR retrotransposons	Gypsy	Romana	290384	292102	1719	70	0.04	8	44	11.43	62.86

Supplemental Table S7 – sRNA counts in the different TE families

				Total sRNA counts					21-nt Counts					24-nt Counts				
Class	Order	Superfamily	Family	n	Average	Min	Max	Sum	Average	Min	Max	Sum	21-nt %	Average	Min	Max	Sum	24-nt %
2	TIR	Mariner	Aison	5	192	65	315	961	110	24	191	550	57.23	50	23	82	252	26.22
1	LTR	Copia	Ale	7	1350	1	4689	9453	309	0	1075	2163	22.88	107	1	354	751	7.94
1	LTR	Copia	Angela	48	3840	62	9879	184338	471	1	1225	22591	12.26	1937	47	4966	92974	50.44
2	TIR	Mutator	Annie	1	10	10	10	10	2	2	2	2	20.00	8	8	8	8	80.00
2	TIR	Mariner	Antonio	1	7	7	7	7	2	2	2	2	28.57	2	2	2	2	28.57
2	TIR	Mutator	Apollo	1	167	167	167	167	51	51	51	51	30.54	77	77	77	77	46.11
2	TIR	Mutator	Argo	3	15	3	26	44	3	0	9	9	20.45	8	3	10	23	52.27
2	TIR	Mutator	Argus	2	57	31	83	114	15	10	20	30	26.32	33	15	51	66	57.89
1	LTR	unknown	Artem	4	205	82	308	821	16	4	26	64	7.80	141	55	208	563	68.57
2	TIR	Mariner	Athos	71	344	1	1330	24401	296	0	1067	21026	86.17	13	0	69	939	3.85
2	TIR	HAT	Aurec	1	6	6	6	6	0	0	0	0	0.00	6	6	6	6	100.00
1	LTR	Gypsy	BAGY	1	494	494	494	494	26	26	26	26	5.26	274	274	274	274	55.47
2	TIR	CACTA	Balduin	4	91	3	207	365	10	0	28	41	11.23	49	2	120	194	53.15
1	LTR	Copia	Barbara	8	438	109	886	3507	49	9	99	393	11.21	295	76	605	2360	67.29
1	LTR	Copia	BARE	5	1065	2	2865	5324	162	1	399	808	15.18	469	0	1227	2346	44.06
2	TIR	unknown	Belus	1	14	14	14	14	6	6	6	6	42.86	2	2	2	2	14.29
1	LTR	Copia	Boba	1	12	12	12	12	2	2	2	2	16.67	10	10	10	10	83.33
2	TIR	CACTA	Boris	3	173	42	251	518	9	2	13	27	5.21	116	31	179	349	67.37
2	TIR	CACTA	Byron	4	359	238	546	1436	43	28	71	172	11.98	236	136	423	945	65.81
2	TIR	CACTA	Cameron	1	0	0	0	0	0	0	0	0	0.00	0	0	0	0	0.00
1	LTR	Gypsy	Carmilla	2	74	73	74	147	3	3	3	6	4.08	37	36	37	73	49.66
2	TIR	CACTA	Caspar	14	423	20	1171	5925	37	2	127	514	8.68	298	13	801	4178	70.51
1	LTR	unknown	Cassandra	8	30	1	44	236	2	0	5	17	7.20	18	1	26	144	61.02
1	LTR	Gypsy	Cereba	2	59	4	114	118	9	0	17	17	14.41	31	1	60	61	51.69
2	TIR	Mutator	Charon	1	85	85	85	85	25	25	25	25	29.41	35	35	35	35	41.18
1	LTR	Copia	Claudia	1	302	302	302	302	29	29	29	29	9.60	185	185	185	185	61.26
2	TIR	CACTA	Clifford	10	1008	9	2236	10080	99	0	246	992	9.84	704	8	1564	7038	69.82
2	TIR	Harbinger	Coeus	1	559	559	559	559	24	24	24	24	4.29	445	445	445	445	79.61
2	TIR	CACTA	Conan	1	1288	1288	1288	1288	132	132	132	132	10.25	897	897	897	897	69.64
1	LTR	Gypsy	Danae	4	127	18	248	509	5	1	8	19	3.73	12	5	17	48	9.43
1	LTR	Copia	Daniela	1	430	430	430	430	34	34	34	34	7.91	97	97	97	97	22.56
1	LTR	Gypsy	Daniela	2	221	30	412	442	12	3	20	23	5.20	48	17	78	95	21.49
2	TIR	Mutator	Deimos	1	179	179	179	179	5	5	5	5	2.79	141	141	141	141	78.77
2	unknown	unknown	Demeter	2	85	83	87	170	4	4	4	8	4.71	63	60	65	125	73.53
1	LTR	Gypsy	Derami	1	327	327	327	327	43	43	43	43	13.15	172	172	172	172	52.60
2	TIR	CACTA	Donald	1	159	159	159	159	30	30	30	30	18.87	81	81	81	81	50.94
1	LTR	Gypsy	Egug	3	237	17	515	711	20	5	48	60	8.44	155	11	351	466	65.54
2	TIR	CACTA	Enac	2	270	256	284	540	18	17	19	36	6.67	211	200	221	421	77.96
2	unknown	unknown	Enuc	1	0	0	0	0	0	0	0	0	0.00	0	0	0	0	0.00
2	TIR	Mariner	Eos	1	91	91	91	91	11	11	11	11	12.09	49	49	49	49	53.85
1	LTR	Gypsy	Erika	7	1164	241	2195	8147	254	48	477	1777	21.81	447	100	912	3129	38.41
1	LTR	Copia	Eugene	4	206	69	313	823	20	12	24	81	9.84	142	49	211	568	69.02
1	LTR	unknown	Eway	1	66	66	66	66	7	7	7	7	10.61	41	41	41	41	62.12
1	LTR	Gypsy	Fatima	32	517	18	1428	16550	50	1	133	1587	9.59	309	8	872	9895	59.79
2	TIR	CACTA	Fergat	1	84	84	84	84	4	4	4	4	4.76	62	62	62	62	73.81
2	TIR	Mariner	Fortuna	4	18	11	29	70	3	1	5	10	14.29	11	6	22	44	62.86
1	LTR	Gypsy	Geneva	1	17	17	17	17	0	0	0	0	0.00	1	1	1	1	5.88
2	TIR	Mutator	Gerald	2	58	19	96	115	17	11	22	33	28.70	33	3	62	65	56.52
1	LTR	unknown	Ginger	1	41	41	41	41	5	5	5	5	12.20	27	27	27	27	65.85
2	TIR	unknown	Gorgon	1	0	0	0	0	0	0	0	0	0.00	0	0	0	0	0.00
1	LTR	unknown	Gujog	5	228	56	486	1142	17	3	45	84	7.36	128	11	290	638	55.87
2	TIR	Mariner	Hades	44	604	1	3342	26596	490	0	2676	21543	81.00	43	0	264	1900	7.14
2	TIR	CACTA	Hamlet	13	408	56	940	5304	40	3	93	517	9.75	287	31	647	3733	70.38
1	LTR	Gypsy	Hawi	1	1693	1693	1693	1693	372	372	372	372	21.97	783	783	783	783	46.25
1	LTR	Gypsy	Heidi	1	14	14	14	14	3	3	3	3	21.43	6	6	6	6	42.86
2	Helitron	Helitron	Helios	1	7	7	7	7	0	0	0	0	0.00	5	5	5	5	71.43
2	TIR	Mutator	Hermes	1	17	17	17	17	0	0	0	0	0.00	17	17	17	17	100.00
2	TIR	CACTA	Heyjude	2	175	149	200	349	16	14	18	32	9.17	83	67	99	166	47.56
1	LTR	Copia	Hopscotch	2	2	1	2	3	0	0	0	0	0.00	0	0	0	0	0.00
1	LTR	Gypsy	HORGY	1	23	23	23	23	9	9	9	9	39.13	8	8	8	8	34.78
2	unknown	unknown	Horus	1	1	1	1	1	0	0	0	0	0.00	1	1	1	1	100.00
2	unknown	unknown	Iapetus	1	7	7	7	7	1	1	1	1	14.29	5	5	5	5	71.43
2	TIR	Mariner	Icarus	74	560	1	2428	41416	323	0	1687	23926	57.77	115	0	333	8529	20.59
1	LTR	Copia	Ida	1	75	75	75	75	9	9	9	9	12.00	55	55	55	55	73.33
1	LTR	Gypsy	Ifis	4	37	22	47	147	2	0	7	8	5.44	26	18	31	105	71.43
1	LTR	Copia	Inga	1	16	16	16	16	0	0	0	0	0.00	11	11	11	11	68.75
2	TIR	CACTA	Isaac	3	604	112	871	1813	16	13	20	49	2.70	356	65	514	1068	58.91
1	Non-LTR	LINE	Isabelle	1	0	0	0	0	0	0	0	0	0.00	0	0	0	0	0.00
2	TIR	Mariner	Jason	4	3	2	3	11	2	2	2	8	72.73	0	0	0	0	0.00
1	LTR	Gypsy	Jeli	4	323	225	408	1293	73	47	108	291	22.51	155	109	206	618	47.80
2	TIR	CACTA	Jorge	7	510	0	1126	3573	40	0	82	279	7.81	280	0	557	1961	54.88
2	TIR	Mutator	Joseph	1	5	5	5	5	0	0	0	0	0.00	4	4	4	4	80.00
2	TIR	CACTA	Jude	5	384	90	1119	1921	45	4	143	227	11.82	245	67	703	1227	63.87
2	TIR	CACTA	Kane	1	15	15	15	15	1	1	1	1	6.67	7	7	7	7	46.67
1	Non-LTR	LINE	Karin	9	173	7	467	1558	22	0	63	202	12.97	122	4	329	1094	70.22
2	TIR	Harbinger	Kerebos	1	70	70	70	70	8	8	8	8	11.43	40	40	40	40	57.14
2	TIR	unknown	Keres	1	0	0	0	0	0	0	0	0	0.00	0	0	0	0	0.00
2	TIR	Harbinger	Kong	11	116	3	318	1281	12	0	28	130	10.15	81	3	225	896	69.95
1	LTR	Gypsy	Latidu	7	1211	237	2582	8478	213	54	566	1494	17.62	627	121	1216	4386	51.73
1	LTR	Gypsy	Laura	18	628	77	1220	11298	154	27	322	2774	24.55	308	21	637	5539	49.03
1	LTR	Gypsy	Lila	1	133	133	133	133	21	21	21	21	15.79	57	57	57	57	42.86
1	LTR	Gypsy	Lisa	2	59	59	59	118	8	8	8	16	13.56	34	34	34	68	57.63
1	LTR	Copia	Liuling	1	1	1	1	1	0	0	0	0	0.00	0	0	0	0	0.00
2	TIR	CACTA	Mandrake	6	62	11	140	372	5	2	10	27	7.26	48	7	105	288	77.42
1	Non-LTR	LINE	Mara	1	2	2	2	2	0	0	0	0	0.00	1	1	1	1	50.00
2	TIR	Mariner	Marius	1	0	0	0	0	0	0	0	0	0.00	0	0	0	0	0.00
1	LTR	unknown	Martin	1	16	16	16	16	1	1	1	1	6.25	15	15	15	15	93.75
1	LTR	Copia	Maximus	3	358	75	561	1074	38	8	59	113	10.52	232	36	359	695	64.71
1	LTR	unknown	Michelle	2	4	4	4	8	0	0	0	0	0.00	2	2	2	4	50.00
2	unknown	unknown	Milton	1	1	1	1	1	0	0	0	0	0.00	0	0	0	0	0.00
2	TIR	Mariner	Minimus	1	18	18	18	18	16	16	16	16	88.89	0	0	0	0	0.00
1	Non-LTR	LINE	Miuse	1	0	0	0	0	0	0	0	0	0.00	0	0	0	0	0.00
2	unknown	unknown	Mordred	1	1	1	1	1	0	0	0	0	0.00	0	0	0	0	0.00
1	Non-LTR	LINE	Morpheus	1	2	2	2	2	0	0	0	0	0.00	1	1	1	1	50.00
2	TIR	Mutator	Murray	1	3	3	3	3	0	0	0	0	0.00	3	3	3	3	100.00
1	Non-LTR	LINE	Nala	2	1	0	1	1	0	0	0	0	0.00	1	0	1	1	100.00
1	Non-LTR	LINE	Nicole	5	8	0	18	38	0	0	0	0	0.00	6	0	15	29	76.32
2	unknown	unknown	Nisos	1	2	2	2	2	2	2	2	2	100.00	0	0	0	0	0.00
1	LTR	Gypsy	Nusif	9	68	17	239	614	9	1	29	84	13.68	23	11	57	205	33.39
2	TIR	Mariner	Oleus	3	22	16	34	66	14	9	16	41	62.12	5	0	15	15	22.73
1	LTR	Copia	Olivia	1	69	69	69	69	5	5	5	5	7.25	54	54	54	54	78.26
1	LTR	Gypsy	Olivia	1	392	392	392	392	39	39	39	39	9.95	104	104	104	104	26.53
1	LTR	Copia	Oref	2	16	5	27	32	2	1	3	4	12.50	9	1	17	18	56.25
2	TIR	Harbinger	Orpheus	5	200	5	341	1001	21	4	44	107	10.69	140	0	258	702	70.13
2	TIR	Mariner	Pan	5	1226	3	2937	6132	735	1	1802	3674	59.92	321	2	718	1606	26.19
1	Non-LTR	LINE	Paula	1	18	18	18	18	2	2	2	2	11.11	13	13	13	13	72.22
2	TIR	CACTA	Pavel	1	121	121	121	121	12	12	12	12	9.92	66	66	66	66	54.55
2	TIR	Mutator	Pilifon	2	2	2	2	4	1	1	1	2	50.00	1	1	1	2	50.00
2	TIR	Mariner	Polyphemus	4	38	0	120	151	10	0	25	41	27.15	21	0	76	83	54.97
1	LTR	Gypsy	Quinta	1	1034	1034	1034	1034	205	205	205	205	19.83	546	546	546	546	52.80
1	LTR	Copia	Rada	3	1	0	2	2	0	0	0	0	0.00	1	0	2	2	100.00
1	Non-LTR	LINE	Ramona	2	7	3	11	14	2	1	2	3	21.43	5	1	9	10	71.43
1	Non-LTR	LINE	Reina	1	2	2	2	2	1	1	1	1	50.00	1	1	1	1	50.00
2	TIR	Mutator	Remus	6	395	129	786	2368	77	29	126	462	19.51	232	42	502	1390	58.70
2	TIR	Mutator	Rhea	1	41	41	41	41	16	16	16	16	39.02	20	20	20	20	48.78
1	LTR	Gypsy	Romana	2	196	176	216	392	12	11	13	24	6.12	102	94	110	204	52.04
1	LTR	Gypsy	Romani	8	183	39	464	1464	16	5	39	128	8.74	113	22	276	907	61.95
1	LTR	unknown	Ronda	1	1	1	1	1	0	0	0	0	0.00	1	1	1	1	100.00
2	TIR	Harbinger	Rong	1	52	52	52	52	3	3	3	3	5.77	33	33	33	33	63.46
1	LTR	Gypsy	Sabrina	28	863	6	1623	24166	86	4	182	2420	10.01	517	1	923	14464	59.85
1	LTR	Gypsy	Sakura	2	552	263	841	1104	69	32	105	137	12.41	325	151	498	649	58.79
1	LTR	Copia	Sasanda	1	542	542	542	542	31	31	31	31	5.72	400	400	400	400	73.80
2	TIR	CACTA	Sherlock	2	15	11	19	30	1	0	1	1	3.33	12	8	15	23	76.67
2	unknown	unknown	Spelt1	1	0	0	0	0	0	0	0	0	0.00	0	0	0	0	0.00
1	Non-LTR	LINE	Stasy	2	930	899	961	1860	114	108	119	227	12.20	607	569	645	1214	65.27
2	TIR	Mariner	Stolos	1	17	17	17	17	9	9	9	9	52.94	5	5	5	5	29.41
1	LTR	Gypsy	Sumana	5	1271	535	2179	6353	333	139	615	1663	26.18	349	153	525	1745	27.47
1	LTR	Gypsy	Sumaya	3	1910	931	2723	5730	472	236	685	1416	24.71	631	264	911	1894	33.05
1	LTR	Gypsy	Surya	1	1170	1170	1170	1170	286	286	286	286	24.44	406	406	406	406	34.70
2	unknown	unknown	Tail	28	2	0	6	50	0	0	2	4	8.00	0	0	2	10	20.00
2	TIR	Mariner	Tantalos	1	1	1	1	1	0	0	0	0	0.00	1	1	1	1	100.00
1	LTR	Copia	TAR1	2	147	100	194	294	13	13	13	26	8.84	102	72	132	204	69.39
2	TIR	CACTA	TAT1	7	293	6	1379	2048	40	0	158	279	13.62	176	5	847	1230	60.06
2	TIR	CACTA	TAT2	1	55	55	55	55	5	5	5	5	9.09	36	36	36	36	65.45
2	TIR	CACTA	TAT3	1	22	22	22	22	4	4	4	4	18.18	12	12	12	12	54.55
2	TIR	CACTA	TAT4	2	6	4	8	12	1	0	1	1	8.33	4	3	5	8	66.67
2	TIR	CACTA	TAT5	1	108	108	108	108	13	13	13	13	12.04	81	81	81	81	75.00
2	TIR	Mariner	Thalos	54	1471	20	6324	79415	1044	2	5395	56368	70.98	232	11	654	12535	15.78
2	TIR	CACTA	Unnamed	1	5	5	5	5	0	0	0	0	0.00	4	4	4	4	80.00
1	LTR	Copia	Unnamed	3	26	0	67	77	2	0	6	6	7.79	19	0	49	57	74.03
1	LTR	Gypsy	Unnamed	1	6	6	6	6	0	0	0	0	0.00	4	4	4	4	66.67
2	TIR	Harbinger	Unnamed	1	64	64	64	64	5	5	5	5	7.81	51	51	51	51	79.69
1	Non-LTR	LINE	Unnamed	14	1	0	6	15	0	0	0	0	0.00	1	0	4	13	86.67
2	TIR	unknown	Unnamed	1	2	2	2	2	1	1	1	1	50.00	1	1	1	1	50.00
2	unknown	unknown	Unnamed	13	92	0	678	1202	8	0	56	101	8.40	66	0	495	856	71.21
2	TIR	Mutator	Vacuna	4	21	9	29	85	3	0	8	12	14.12	14	9	19	55	64.71
1	LTR	unknown	Varna	1	237	237	237	237	8	8	8	8	3.38	183	183	183	183	77.22
1	LTR	unknown	Veju	2	711	126	1296	1422	47	10	83	93	6.54	544	83	1004	1087	76.44
2	TIR	Harbinger	Victor	1	10	10	10	10	2	2	2	2	20.00	7	7	7	7	70.00
2	TIR	CACTA	Vincent	2	39	0	77	77	5	0	9	9	11.69	28	0	55	55	71.43
2	unknown	unknown	Walter	1	138	138	138	138	15	15	15	15	10.87	100	100	100	100	72.46
1	Non-LTR	LINE	Wendy	1	0	0	0	0	0	0	0	0	0.00	0	0	0	0	0.00
1	LTR	Gypsy	WHAM	19	963	263	1995	18293	85	20	254	1611	8.81	601	175	1282	11420	62.43
1	LTR	Gypsy	Wilma	8	210	65	581	1681	31	8	97	247	14.69	114	32	319	915	54.43
1	LTR	Copia	WIS	26	3578	123	6594	93023	358	14	677	9307	10.01	1973	84	3759	51295	55.14
2	unknown	unknown	Xabor	1	37	37	37	37	0	0	0	0	0.00	31	31	31	31	83.78
1	LTR	unknown	Xalax	2	152	113	190	303	9	4	14	18	5.94	109	90	128	218	71.95
2	unknown	unknown	Xanti	2	19	0	38	38	2	0	3	3	7.89	15	0	29	29	76.32
2	unknown	unknown	XC	4	96	4	304	385	9	0	28	35	9.09	69	3	214	275	71.43
2	TIR	Harbinger	Xenon	3	17	1	38	52	2	0	6	7	13.46	11	1	24	32	61.54
2	unknown	unknown	Xenos	9	37	4	109	336	2	0	4	15	4.46	29	1	91	262	77.98
2	unknown	unknown	XG	1	0	0	0	0	0	0	0	0	0.00	0	0	0	0	0.00
2	unknown	unknown	Xi	2	28	20	35	55	2	0	3	3	5.45	18	11	24	35	63.64
2	unknown	unknown	Xitos	3	21	13	33	64	1	0	2	2	3.13	19	11	28	56	87.50
2	unknown	unknown	Xusag	1	10	10	10	10	0	0	0	0	0.00	7	7	7	7	70.00
1	LTR	Gypsy	Yasmin	1	1	1	1	1	1	1	1	1	100.00	0	0	0	0	0.00
1	Non-LTR	LINE	Yvonne	1	5	5	5	5	1	1	1	1	20.00	3	3	3	3	60.00
2	TIR	Mutator	Zeus	1	30	30	30	30	2	2	2	2	6.67	19	19	19	19	63.33
1	LTR	Gypsy	Zuhra	1	1	1	1	1	0	0	0	0	0.00	1	1	1	1	100.00

Supplemental Table S8 – Estimates of LTR age of insertion and cytosine methylation in the CG, CHG, and CHH contexts

			Transition frequency (%)			
**Element**	**MYA**	**±SD**	**CG**	**CHG**	**CHH**	**CG-CHG-CHH**
TtLaura1	0.81	0.15	3.11	3.19	0.56	0.63
TtAngela1	3.50	0.48	10.16	17.59	3.57	3.75
TtAngela3	2.58	0.41	9.09	19.57	1.29	1.95
TtAngela4	2.02	0.36	9.14	14.05	0.63	0.99
TtFatima1	4.27	0.99	6.12	7.50	1.27	2.40
TtMartin1	1.44	0.59	5.88	9.09	3.01	2.96
TtWis2	1.55	0.31	5.91	10.19	0.66	1.30
TmAngela4	1.89	0.35	7.25	14.00	1.33	1.47
TmAngela7	1.04	0.25	5.32	9.35	0.00	0.36
TmAngela8	4.81	0.56	13.09	24.04	3.14	3.46
TmAngela9	2.45	0.40	6.70	11.61	0.97	1.31
TmAngela12	2.24	0.38	7.69	12.84	1.86	2.71
TmAngela13	0.67	0.20	3.19	5.66	0.19	0.37
TmAngela14	2.06	0.36	6.63	12.75	0.68	0.99
TmAngela15	0.96	0.30	2.94	5.26	1.25	1.54
TmErika1	1.73	0.21	4.60	6.78	0.98	1.48
TmFatima1	3.38	0.88	6.00	7.14	1.95	3.11
TmJeli1	2.56	0.71	8.62	11.63	1.10	2.13
TmJeli2	2.96	0.77	3.70	4.55	3.83	3.70
TmRom1	2.45	0.31	8.56	13.59	0.83	1.59
TmRom2	2.94	0.32	5.25	9.28	4.45	4.99
TmTari1	6.33	0.86	5.33	7.92	7.67	10.48
TmWham1	4.17	0.57	15.38	17.48	2.61	2.92
TmWham2	4.60	0.62	18.02	22.99	2.46	2.75

MYA= time of insertion in million year; SD= MYA standard deviation; in element name: Tm = *T. monococcum*, Tt = *T. turgidum*.
